# Chronic inflammation does not mediate the effect of adiposity on grip strength: results from a multivariable Mendelian randomization study

**DOI:** 10.1038/s41598-023-43908-y

**Published:** 2023-10-06

**Authors:** Tom Norris, Eleanor Sanderson, Rachel Cooper, Victoria Garfield, Snehal M. Pinto Pereira

**Affiliations:** 1 Division of Surgery and Interventional Science, Faculty of Medical Sciences, Institute of Sport, Exercise and Health, UCL, London, UK; 2 Population Health Sciences, Bristol Medical School, University of Bristol, Bristol, UK; 3 AGE Research Group, Faculty of Medical Sciences, Translational and Clinical Research Institute, Newcastle University, Newcastle, UK; 4 NIHR Newcastle Biomedical Research Centre, Newcastle University and Newcastle Upon Tyne NHS Foundation Trust, Newcastle Upon Tyne, UK; 5 MRC Unit for Lifelong Health and Ageing at UCL, Institute of Cardiovascular Science, University College London, London, UK

**Keywords:** Epidemiology, Genetics research, Risk factors

## Abstract

The relationship between adiposity and grip strength (GS) is complex. We investigated whether one pathway through which adiposity affects GS was via chronic inflammation. 367,583 UK Biobank participants had body mass index (BMI), waist-hip-ratio (WHR), C-reactive protein (CRP) and GS data. Univariable Mendelian randomization (MR) and multivariable Mendelian randomization (MVMR) analyses (using inverse variance weighted (IVW) weighted median estimates (WME) and MR-Egger models) estimated total, direct and indirect effects of adiposity traits on GS using genetic instruments for BMI and WHR (exposures) and CRP (mediator). Observational findings suggested higher BMI was associated with stronger grip, e.g., in males, per standard deviation (SD) higher BMI, GS was higher by 0.48 kg (95% confidence interval(CI):0.44,0.51), independent of CRP. For males MR estimates were directionally consistent; for females, estimates were consistent with the null. Observational findings for WHR suggested that higher WHR was associated with weaker grip. In multivariable MR-IVW analyses, effects in males were consistent with the null. In females, there were consistent effects such that higher WHR was associated with stronger grip, e.g., 1-SD higher WHR was associated with 1.25 kg (MVMR-Egger; 95% CI:0.72,1.78) stronger grip, independent of CRP. Across sexes and adiposity indicators, CRP’s mediating role was minor. Greater adiposity may increase GS in early old age, but effects vary by sex and adiposity location. There was no evidence that inflammation mediated these effects.

## Introduction

Muscle weakness is a component of several important age-related conditions including sarcopenia and frailty^1–3^. At the population level it is usually proxied by low absolute values of grip strength, and it is associated with functional decline, fractures, mobility disability, loss of independence and all-cause mortality^4–9^. Against the backdrop of an ageing population^10^, the disease and disability burden associated with muscle weakness represents a major public health concern^11–13^.

Several genetic and environmental factors over the life course have been associated with grip strength across adulthood^2, 14^. In particular, the relationship between adiposity and grip strength has been the focus of a number of observational studies, with findings suggesting a complex relationship^15, 16^ that may differ between sexes^17, 18^, across age^18, 19^ and indicators of adiposity^16, 18^. Attempting to disentangle the causal effects of adiposity on grip strength, we recently performed the first Mendelian Randomization (MR) study, using genetic and phenotypic data from over 400 k participants in the UK Biobank (UKB). MR uses genetic variation to answer questions about whether modifiable exposures, e.g., adiposity, influence outcomes, e.g., grip strength. When underlying assumptions are valid, causality can be assumed. For an up-to-date overview of MR principles and practices, see Sanderson et al.^20^. In our MR study, we provided evidence to suggest that higher body fat percentage (BF%, both sexes) and waist-hip ratio (WHR, males only) were causally related to lower grip strength at older ages^21^. Given worldwide epidemic levels of obesity^22^, unpicking the aetiological mechanism underpinning the role of adiposity on strength is crucial. Furthermore, as ageing is associated with a redistribution of adipose tissue (e.g., progressive loss of subcutaneous fat and an accumulation of visceral and ectopic fat^23^), it is important to elucidate whether any proposed mechanistic effect is universal across different adiposity traits or whether effect-mechanism heterogeneity exists.

One viable pathway via which adiposity may influence strength involves the obesity-related state of chronic low-grade inflammation, as indicated by inflammatory markers including C-reactive protein (CRP). Obesity, typically assessed by higher body mass index (BMI), is causally related to circulating levels of CRP^24^ which, in turn, is associated with reduced muscle mass^25, 26^, strength^25, 26^ and power^27^. Stenholm and colleagues^15^ investigated the mediating role of CRP in the relationship between obesity (BMI > 30 kg/m^2^) and very low hand grip strength in 2,021 adults aged ≥ 55 years. They observed that a longer duration of obesity was associated with increased odds of very low grip strength and higher CRP levels; higher CRP was correlated with lower hand grip strength. However, the study was cross-sectional, obesity duration was based on self-report and there was potential over-adjustment for body weight^28^. Therefore, drawing conclusions, including regarding the temporal ordering of relationships, was difficult.

Considering current knowledge gaps and methodological limitations, we performed a Multivariable Mendelian Randomization (MVMR) analysis, in which genetic instruments for several phenotypes are used to estimate mediating effects, in an attempt to understand one possible mechanism via which adiposity may causally affect grip strength. We used genetic instruments for BMI, WHR and CRP to assess the total and direct causal effects of general (BMI) and central (WHR) adiposity on grip strength and to identify whether any effect operates via an inflammatory (CRP) pathway. To increase robustness of our findings and triangulate evidence obtained across different analytical frameworks which are subject to distinct assumptions, we also performed a complementary observational mediational analysis using phenotypic measures of BMI, WHR, CRP and grip strength. Due to evidence of sex-differences in adiposity-grip strength associations^17^, and marked sex-differences in grip strength^29^, we chose a-priori to run all analyses stratified by sex.

## Results

Males had a higher mean grip strength than females (mean (standard deviation (SD)): 42.1 kg (8.8) males; 25.3 kg (6.3) females). Mean BMI and WHR were also higher in males, whereas females had higher levels of CRP (Table 1).

**Figure 1:**
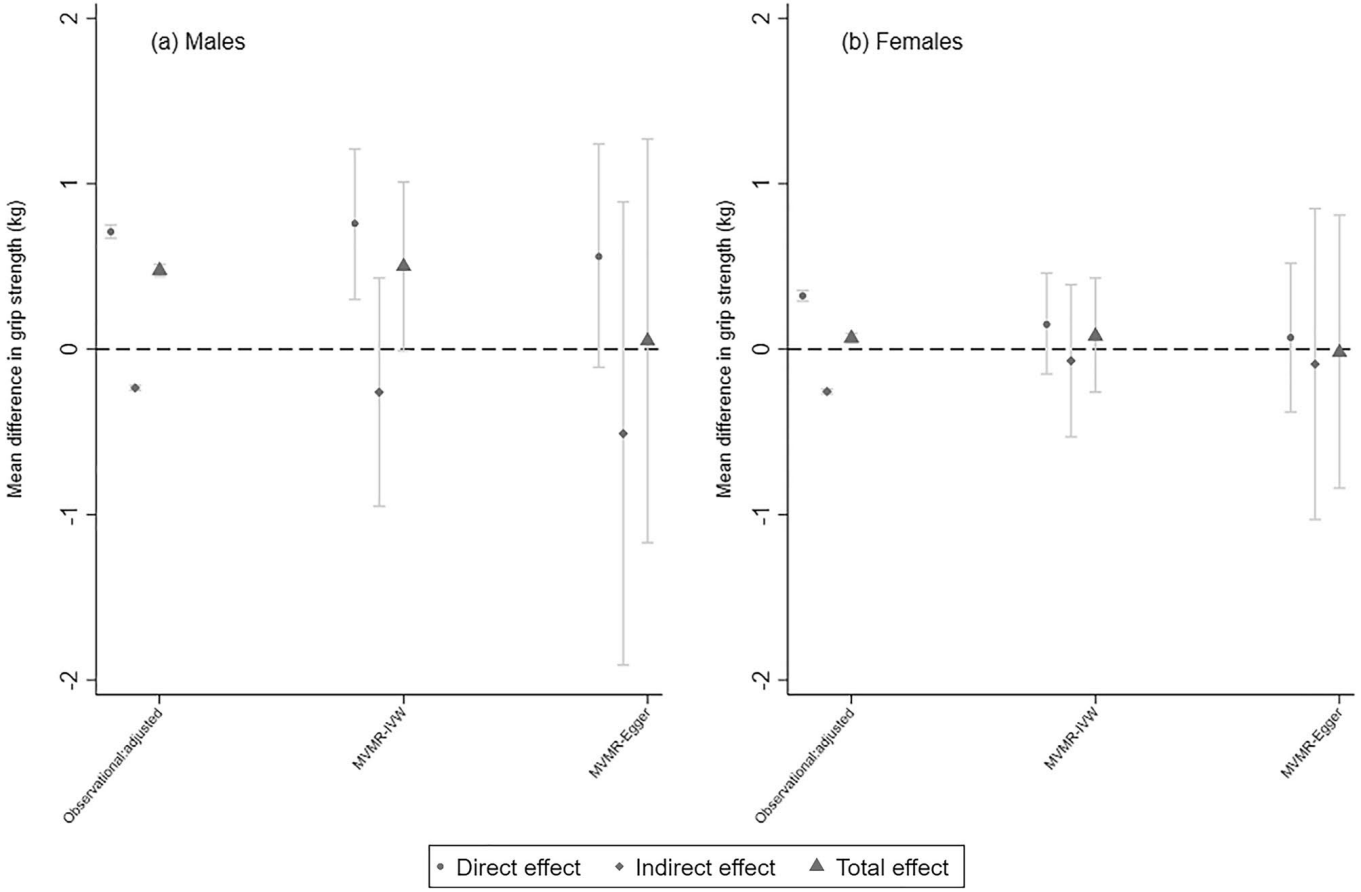
Observational and MR estimates of direct, indirect and total effects between BMI and GS.

**Table 1:** Sample characteristics (*n* = 367,583).

Variable^a^	Males (170, 175)	Females (197, 408)
Sociodemographic characteristics		
Age at recruitment (years)	57.0 (8.1)	56.5 (7.9)
Townsend deprivation index^b^	− 2.4 (− 3.8, 0.0)	− 2.4 (− 3.8, − 0.1)
Currently smoking		
No	150, 659 (80.5)	180, 883 (91.6)
Yes	19, 516 (11.5)	16, 525 (8.4)
Alcohol consumption		
Less than daily	124, 797 (73.3)	163, 571 (82.9)
Almost/daily	45, 378 (26.7)	33 837 (17.1)
Physical activity		
Active (vigorous activity ≥ 4x/wk)	38, 564 (22.7)	29, 569 (15.0)
Inactive (vigorous activity < 4x/wk)	131, 611 (77.3)	167, 839 (85.0)
Arthritis		
No	158, 211 (93.0)	175, 488 (88.9)
Yes	11, 964 (7.0)	21, 920 (11.1)
Asthma		
No	151, 997 (89.3)	173, 268 (87.8)
Yes	18, 178 (10.7)	24, 140 (12.2)
Depression		
No	163, 083 (95.8)	184, 010 (93.2)
Yes	7, 092 (4.2)	13, 398 (6.8)
Physical characteristics		
BMI (kg/m^2^)	27.3 (25.0, 30.0)	26.0 (23.4, 29.5)
Waist-hip ratio	0.9 (0.1)	0.8 (0.1)
C-reactive protein (mg/L)	1.3 (0.7, 2.5)	1.4 (0.7, 2.9)
Grip strength (kg)^c^	42.1 (8.8)	25.3 (6.3)

^a^continuous variables summarised as either mean(SD) or median(25th, 75th centile), categorical variables as N(%).

^b^a higher index indicates more deprivation.

^c^Maximum of left and right hand measure.

## *BMI-*grip strength

### Observational analysis

In males, higher BMI was associated with greater grip strength, e.g., in confounder-adjusted models, a 1 SD higher BMI was associated with a 0.48 kg stronger grip (95% CI: 0.44, 0.51). This total effect was decomposed into a direct effect which was even larger than the total effect (*β* = 0.71 kg; 95% CI: 0.67, 0.75) alongside a negative indirect effect via CRP which reduced grip strength (*β* =   − 0.23 kg; 95% CI: − 0.25, − 0.22) (Fig. 1 and Supplementary Table 4). A similar pattern was observed in females, though effect sizes were smaller.

**Figure 2:**
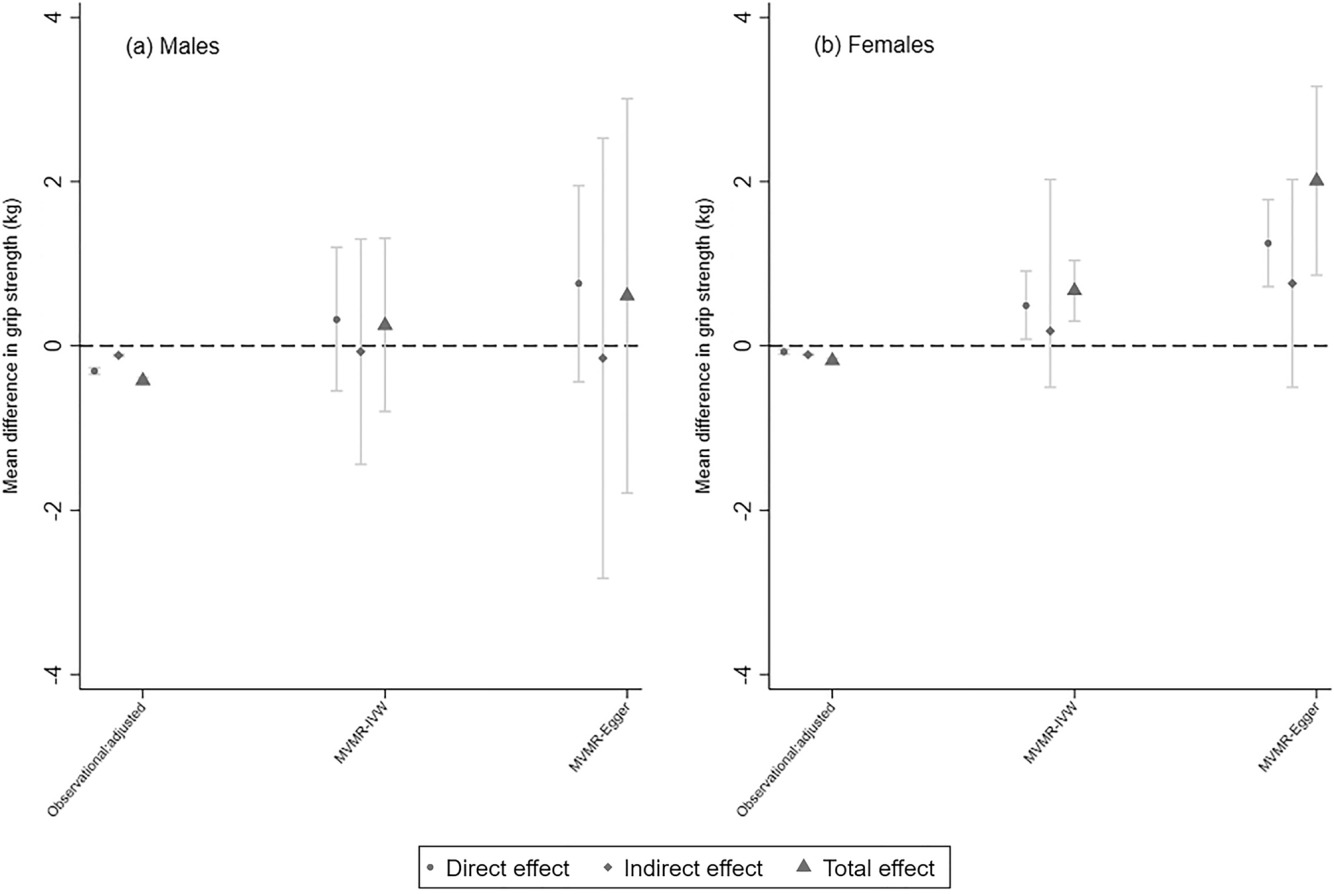
Observational and MR estimates of direct, indirect and total effects between WHR and GS.

### Genetic analysis

#### Total effect (univariable Mendelian randomization (MR))

Total effect estimates from univariable MR-IVW (Mendelian randomization inverse variance weighted) were directionally consistent with those from observational analyses, suggesting higher BMI is associated with stronger grip (Fig. 1 and Supplementary Table 5). Similar to the observational analysis, estimates were higher in males than females, e.g., a 1 SD higher BMI was associated with a 0.50 kg (95 CI: − 0.01, 1.01) stronger grip in males compared to 0.08 kg (95% CI: − 0.26, 0.43) in females. Furthermore, confidence intervals for associations in females suggested a total effect consistent with the null.

#### Direct & indirect effects (multivariable MR (MVMR))

Estimates of the direct and indirect effects of BMI on grip strength, after accounting for the mediating role of CRP, obtained from the MVMR-IVW (multivariable Mendelian randomization inverse variance weighted) are displayed in Fig. 1 and in Supplementary Table 6. Conditional F-statistics are provided in Supplementary Table 6 and show that the single nucleotide polymorphisms (SNPs) included in the analysis are strong instruments for assessing the direct effects of BMI while accounting for CRP (F > 20 in both sexes). Q_A_ (generalised versions of Cochran’s Q) statistics were, however, greater than the number of included SNPs (N_BMI_ = 164) (Supplementary Table 6), indicating excess heterogeneity and the potential for pleiotropy. As such, we also present direct effects of adiposity after Q_A_-statistic minimisation (Q-het) to account for weak instruments and high heterogeneity.

In general, patterns of associations from the MVMR analyses were broadly comparable to those from observational analyses. In males, while the total effect of BMI on grip strength (from univariable MR-IVW), was 0.50 kg (95% CI: − 0.01, 1.01) per 1 SD increase in BMI (Fig. 1 and Supplementary Table 6), the direct effect of BMI on grip strength from the corresponding MVMR-IVW was greater at 0.76 kg per 1 SD increase in BMI (95% CI: 0.30, 1.21). Similarly, and in line with observational estimates, the indirect effect beta estimates were negative in the MVMR-IVW analyses, however confidence intervals were wide and straddled the null.

In females, the size of the direct effect (MVMR-IVW: *β* =  0.15; 95% CI: − 0.15, 0.46) was also greater than the total effect (*β* =  0.08; 95% CI: − 0.26, 0.43). However, unlike males, the effect sizes for the direct, indirect and total effects and their corresponding confidence intervals all suggested an effect of BMI on grip strength which was consistent with the null.

#### Sensitivity analyses

For the total effect of BMI on grip strength, in general, consistent patterns of associations to those reported above were observed for both sexes in the MR-WME (Mendelian randomization weighted median estimator) and MR-Egger (Mendelian randomization Egger) analyses with confidence intervals continuing to straddle the null (except for the WME analyses in males; Supplementary Table 5). For both sexes, MR-Egger intercept terms (Supplementary Table 5) and funnel plots (Supplementary Figs. 2 & 3) did not suggest the presence of horizontal pleiotropy; removing SNPs associated with confounders did not have a substantive impact on findings (Supplementary Table 7).

For the direct effect of BMI on grip strength in males, a similar sized effect was observed in MVMR-Egger (*β* =  0.56; 95% CI: − 0.11, 1.24) and the *Q*_het_ analysis (*β* =  0.61; 95% CI: − 0.16, 1.15) and confidence intervals included the null (Supplementary Table 6). In females, direct and indirect effect estimates in MVMR-Egger and *Q*_het_ were consistent with the null.

### WHR-grip strength

#### Observational analysis

In both sexes, there was a negative association between WHR and grip strength, such that higher WHR was associated with lower grip strength, e.g., for males in confounder-adjusted models, a 1 SD higher WHR was associated with a − 0.42 kg (95% CI: − 0.46, − 0.39) lower grip strength (Fig. 2 and Supplementary Table 4). After accounting for the effect of WHR through CRP, the direct effect on grip strength remained negative, but weaker than the total effect e.g., for males the direct effect was − 0.31 kg (95% CI: − 0.35, − 0.27).

### Genetic analysis

#### Total effect (univariable MR)

In general, patterns of associations from the univariable (and MVMR) analyses were inconsistent with those from observational analyses. For example, a 1 SD increase in WHR was associated with a 0.25 kg (95% CI: − 0.80, 1.31) and 0.67 kg (95% CI: 0.30, 1.04) stronger grip in males and females, respectively (supplementary table 5).

#### Direct & indirect effects (multivariable MR)

Estimates of the direct and indirect effects of WHR on GS, after accounting for the mediating role of CRP, obtained from the MVMR-IVW analysis, are displayed in Fig. 2 and in Supplementary Table 6. Q_A_ statistics were greater than the number of included SNPs (N_WHR_ = 112), indicating excess heterogeneity and potential pleiotropy. Conditional F-statistics show that the WHR instrument, accounting for the effect of CRP, was of adequate strength in females (F = 12.04) but was weaker in males (F = 6.05; Supplementary Table 6). As such, we also present direct effects of adiposity after Q_A_-statistic minimisation (Q_-het_) to account for weak instruments and high heterogeneity.

**Figure 3:**
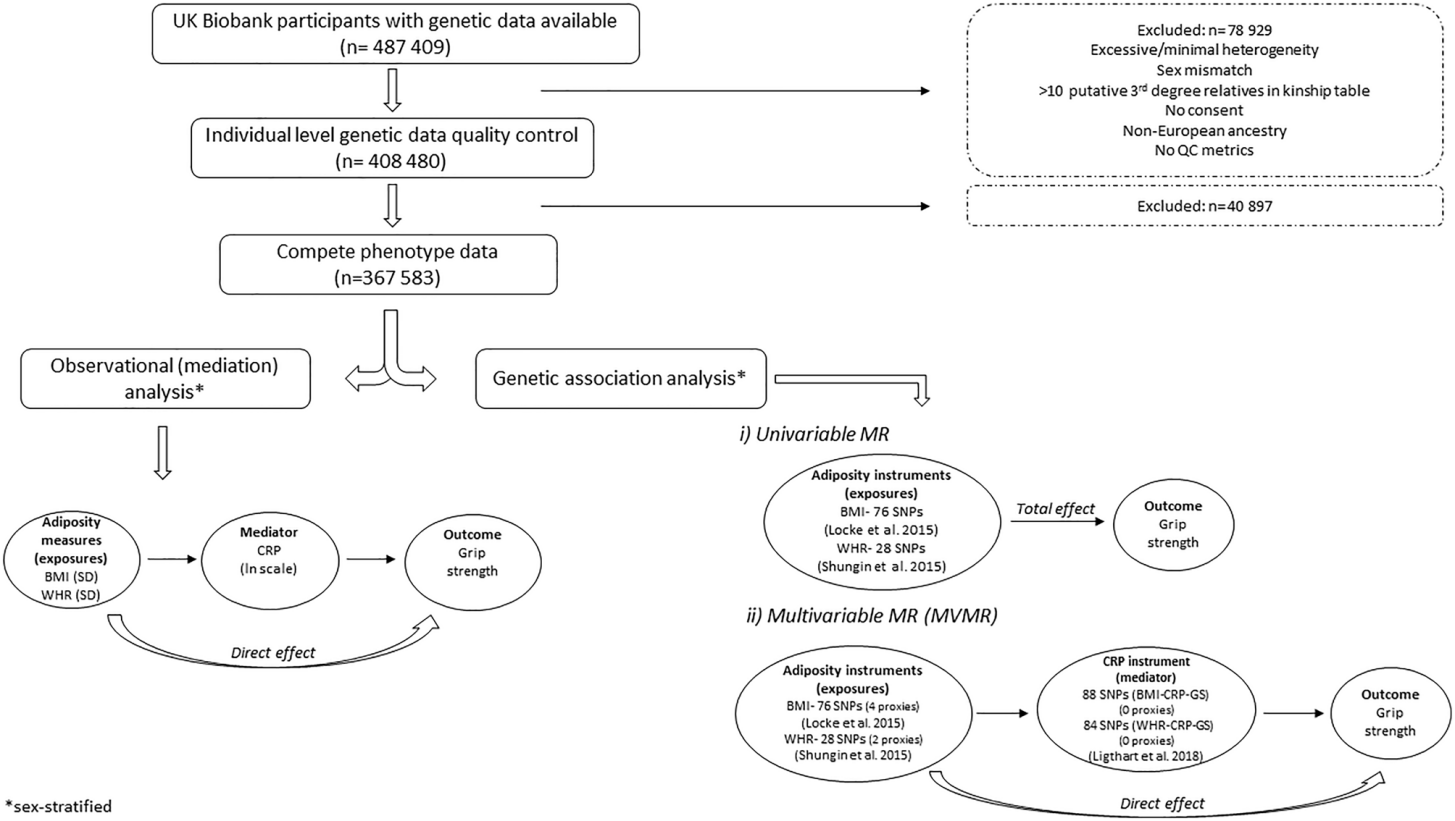
Sample flow diagram and study design illustrating mediation approach.

For WHR in males, estimates of the direct, indirect and total effects and their corresponding confidence intervals suggested an effect of WHR on grip strength which was consistent with the null (Fig. 2 and Supplementary Table 6). In females however, the direct effect suggested that a 1 SD higher WHR was associated with 0.49 kg (95% CI: 0.08, 0.91) stronger grip. Whilst the indirect effect beta estimate was positive, the confidence interval was wide and straddled the null.

#### Sensitivity analyses

For the total effect of WHR on GS, the sensitivity analyses in males produced directionally inconsistent estimates with wide confidence intervals which straddled the null; there was no evidence of horizontal pleiotropy (Supplementary Fig. 4, Supplementary Tables 6 & 9). For direct and indirect effects of WHR in males, sensitivity analyses provided similar findings to those observed in the main MVMR-IVW analysis (Supplementary Tables 6 & 9).

For females, whilst the MR-WME (*β* =  0.94; 95% CI: 0.56, 1.31) and MR-Egger (*β* =  2.01; 95% CI: 0.86, 3.16) produced consistent estimates to those obtained from MR-IVW, there was evidence of horizontal pleiotropy (Supplementary Fig. 5 & Supplementary Table 5). When analyses were re-run after removing (1) six SNPs associated with confounders and (2) three SNPs of high influence, estimates of the total effect of WHR from MR-IVW, MR-WME and MR-Egger all still indicated that higher WHR was associated with stronger grip strength (Supplementary tables 7 & 8). The direct effect from the MVMR-Egger analysis was consistent with (but larger than) the direct effect observed in the MVMR-IVW analysis (*β* =  1.25 kg, 95% CI: 0.72, 1.78) (Supplementary Table 6). However, evidence of horizontal pleiotropy was observed (*p*_intercept_ < 0.001), which persisted even after removing the WHR SNPs which were associated with confounders and exerted high influence (Supplementary Tables 9 & 10). Moreover, the direct effect of WHR was maintained in the *Q*_het_ analysis: *β* =  0.52 (95% CI: 0.11, 0.85) (supplementary table  6).

## Discussion

We utilised genetic and phenotypic data from 367,583 individuals in several complementary analyses (one observational and two (or more) different MR analyses) to investigate causal links between adiposity and grip strength. We also aimed to determine whether any effects operate independently of the mediating effects of chronic inflammation. In males, results from observational and genetic analyses generally agreed, showing higher BMI was associated with greater grip strength independently of CRP. However, for WHR observational and genetic analyses were inconsistent, but both MR analyses indicated no association with grip strength. In females, while patterns of associations between BMI and grip strength in observational and genetic analyses were broadly consistent, the MR analyses effect sizes and corresponding confidence intervals all spanned the null. For WHR, while observational and MR analyses were inconsistent, the two MR analyses demonstrated that higher WHR was associated with stronger grip and this was independent of the mediating effect of CRP.

Our finding of a direct effect of higher BMI on greater grip strength in males was robust to several sensitivity analyses. That this direct effect, which is independent of the mediating role of CRP, was greater than the total effect, underlines the importance of investigating the (many) pathways via which BMI is related to grip strength. Thus our observation that the effect of BMI on grip remains important even after accounting for inflammation agrees with findings from Stenholm et al.^15^. However, in contradiction to Stenholm and colleagues who observed that the direct effect of longer obesity duration was associated with lower grip strength, here, we found that, on average over a lifetime, the direct effect of BMI resulted in stronger grip at early older ages. Possible explanations for discrepant findings include the fact that Stenholm et al. adjusted for current weight and so their results could be explained by the positive association between height and grip strength^28^. Differences could also be attributed to the average age of participants in the two studies. Our sample (average age: 57 years) was approximately 10 years younger than those examined by Stenholm et al. In our analysis, both observational and multiple MR estimates indicated that higher BMI was associated with stronger grip in males, it may be that the anabolic effects of fat on muscle, at early older ages (observational estimates) and on average over the lifetime (MR estimates), are outweighing the catabolic effects thought to lead to sarcopenia later in life, especially among males^18^.

Our observation of a positive average lifetime effect of BMI on grip in males reported here agrees with our previous MR analysis in UKB participants^21^ and is also supported by previous work in other cohorts adopting different analytical approaches^17^. Of note, is that in the current work we used a different genetic instrument for BMI to that used previously^21^. The consistency of findings across multiple analysis methods, datasets and use of different instruments (here and in our previous MR analysis^21^), strengths the basis for a causal interpretation of the BMI-grip strength relationship in men at early old age. Importantly, we extend our previous work by showing that even after accounting for any mediating effect of chronic inflammation, a relationship between BMI and grip strength remains in males. However, as our MR analyses estimate life-long effects of the BMI-CRP pathway on grip strength, we are unable to speculate as to the timing and nature of the relationship between these phenotypes, which we know vary over the life course^29–31^. For example, analyses of serial BMI and grip strength data from the same individuals over time, suggests a changing relationship between BMI and grip strength as the life course progresses^19, 32^.

In females, our MR analyses revealed no effect of BMI (total or direct) on grip strength. This difference between males and females has been observed elsewhere^17, 18^ and may reflect sex differences in body composition, with males tending to have, on average, a higher percentage of lean mass and a more favourable lean: fat mass ratio^33^. Relatedly, findings between sexes could also reflect the inability of BMI to differentiate between lean and fat mass and the fact that at a population level, BMI in males might be more representative of lean mass whereas in females, it may be more representative of fat mass. It may also be a result of sex differences in the anabolic response to mechanical loading. Fat mass (captured by BMI) acts as a mechanical load that promotes muscle growth and function^34^. This anabolic response is usually greater in males due to higher circulating levels of testosterone^35, 36^, which may contribute to observed sex differences.

Our consistent findings in genetic analyses showing higher WHR being associated with stronger grip in females is surprising. Although this agrees with our previous MR findings ^21^, it differs to our observational analysis. While two previous observational studies noted higher waist circumference was associated with stronger grip, these associations reversed in direction when adjusted for BMI^16, 17^. Abdominal obesity, as indicated by a high WHR, is known to produce high amounts of inflammatory cytokines and adipokines^37^ such as adiponectin, tumour necrosis factor-α (TNF-α), and interleukin-6 (IL-6)^38^. Adipose tissue-derived IL-6 drains into the hepatic portal system, promoting the production of CRP in the liver^39–41^. At the cellular level, CRP has been associated with decreased protein synthesis^42^, muscular degradation^43^ and loss of muscle mass^25^. It is therefore surprising that our study suggests higher WHR is associated with stronger grip in females and furthermore, that there was no evidence for an indirect effect operating via CRP, which is associated with reduced muscular strength^26, 44^.

A major study strength is the complementary analyses (one observational and several different MR analyses) that we employed to investigate causal chains of associations between adiposity, inflammation, and strength. For example, using an MVMR approach, we have been able to establish the effects of adiposity on grip strength which operate independently of CRP. The genetic variants we used as instruments are inherited at conception between parents and offspring randomly, this reduces the likelihood that our MR estimates are affected by residual confounding and reverse causality^45^. That these genetic variants were obtained from large, genome wide association studies (GWAS) that did not include UK Biobank participants (which we used to derive SNP-GS estimates), is a further strength as it removes the risk of introducing bias from sample overlap. Importantly, by performing both observational and multiple MR analyses (that have different strengths and underlying assumptions) and via the use of multiple indicators of adiposity, we have also been able to triangulate findings to comprehensively explore the relationship between adiposity and GS. Within our MR approach, we employed three different models (IVW, MR-Egger and WME) to estimate the *total* effect of adiposity on grip strength and two (MVMR-IVW, MVMR-Egger) to estimate the *direct* effect. We also repeated MR analyses after removing SNPs associated with confounders or that exerted a high influence on estimates. Furthermore, the use of the Q-minimisation procedure in MVMR analyses enabled us to obtain more robust causal estimates in the presence of weak instruments and/or high heterogeneity (indicative of horizontal pleiotropy). The general agreement across the different MR models, and more broadly with our observational findings and the wider literature, especially for the effects of BMI in males, strengthens the causal interpretation of our findings.

We also acknowledge some study limitations. Another adipokine, IL-6, has been implicated in the development of sarcopenia and declines in physical function, with findings from observational studies of individuals aged > 75 years showing higher levels of IL-6 associated with reductions in muscular strength^26, 46^. While GWAS of IL-6 exist^47, 48^, we decided not to consider IL-6 as a potential mediator due to concerns about the validity of this instrument (e.g., adjustment for BMI and number of SNPs). In attempting to mitigate the risk of sample overlap, we included genetic variants obtained from GWAS excluding UKB participants. This resulted in a smaller number of SNPs in our instruments than otherwise would have been possible, which likely reduced the power of our MR analyses and may have contributed to some weak instrument bias e.g., for the WHR instrument in males. Thus, estimates of the direct effect of WHR on GS in males should be interpreted with caution. Whilst grip strength is a convenient and commonly used proxy for overall body strength and the Jamar dynamometer has good reliability and reproducibility^49^, grip strength is a measure of upper limb strength. Evidence is mixed with regard to whether grip strength is an adequate proxy for overall muscle strength^50, 51^ and relationships between adiposity and strength are likely to vary by location of strength measurement. Finally, selection bias into UKB is evident^52^. This has to the potential to induce collider bias^53^ and bias estimates from both observational and MR analyses^54^.

As a consequence of worldwide population ageing, younger generations are likely to spend greater proportions of their (longer) lives living with age-related conditions characterised by muscle weakness, compared to older generations. In addition, the current epidemic levels of obesity necessitate a comprehensive understanding of how adiposity is related to muscle strength. We have shown that on average over a lifetime greater adiposity may increase grip strength in early old age, but the effects vary by sex and adiposity location (i.e., total vs central). Nonetheless, across both sexes and adiposity indicators, inflammation (proxied by CRP) did not play a major mediating role. Therefore, future studies are warranted to identify alternative mechanisms (including other markers of inflammation) via which adiposity affects muscle strength (e.g., via insulin resistance^15^ and/or reduced physical activity).

## Methods

### Study participants

UK Biobank (UKB; https://www.ukbiobank.ac.uk/), described in detail elsewhere^55^, is a large, prospective cohort of individuals aged 40–69 years at recruitment (2006–2010) from across the UK^55^. The sample examined here included 367,583 European ancestry participants with available data on genotypes and all relevant phenotypes (details in Fig. 3). Participants provided informed consent; ethical approval was given by the North-West Multicentre Research Ethics Committee. All methods and data collection procedures were performed in accordance with relevant guidelines and regulations including the Declarations of Helsinki.

### Exposure: adiposity measures

Adiposity measures were obtained at baseline following standardised protocols^56^: weight was measured using a Tanita BC-418 MA body composition analyser; height measured with a Seca-202 height measure; waist and hip circumferences measured using a Seca-200 tape measure. BMI (kg/m^2^) and WHR were derived.

### Mediator: C-reactive protein

CRP (mg/L) was measured by immunoturbidimetric-high sensitivity analysis on a Beckman Coulter AU5800. Detection limits were between 0.08 and 80 mg/L^57, 58^.

### Outcome: grip strength

Grip strength was assessed using a Jamar J00105 hydraulic hand dynamometer. Participants sat upright in a chair with their forearms on armrests. They were asked to squeeze the dynamometer’s handle as hard as they could with their right hand for about 3 s. The grip strength measurement was then repeated using the same protocol for the left hand^59^. We examine the maximum recorded value (greater than 0) from either hand.

### Potential confounders

Potential confounders were identified from a directed acyclic graph (Supplementary Fig. 1). They included: the Townsend index^60^ (a measure of area-level deprivation), smoking status, physical activity, alcohol intake, age at recruitment, and comorbidities (arthritis, asthma and depression) (details in Supplementary material).

### Genetic instrument selection

(i)Adiposity

We used 76 and 28 near-independent SNPs (Linkage Disequilibrium (LD) r^2^ < 0.001, clumping window > 250 kb) for BMI and WHR which achieved genome-wide significance (*p* < 5 × 10^−8^) in the respective (sex-combined) genome-wide association studies (GWAS)^61, 62^. Whilst SNP selection was based on significance in sex-combined GWAS, sex-specific summary statistics were available for both instruments and these were extracted. Instrument details are provided in Supplementary Tables 1 & 2. SNPs were selected from GWAS which did not include UKB participants. Instrument F-statistics, obtained from regressions of each phenotype on the respective SNPs, were 46.8 (males) and 45.7 (females) for BMI and 26.4 (males) and 81.5 (females) for WHR; the variance explained was 2.1% (males) and 1.7% (females) for BMI and 0.4% (males) and 1.1% (females) for WHR.

 (ii) CRP

We used 88 (in BMI-CRP-grip strength MVMR) and 84 (in WHR-CRP-grip strength MVMR) near-independent SNPs achieving GWA significance (*p* < 5 × 10^−8^) in the recent sex-combined GWAS conducted by Ligthart et al.^63^, which comprised > 200,000 European individuals from 88 studies, but did not include UKB (further instrument details in Supplementary Tables 1 & 2). See ‘*MVMR’* below for explanation regarding different number of SNPs used. The F-statistic and variance explained of the instruments were 30.5 (males) and 55.4 (females) and 1.6% (males) and 2.4% (females), respectively.

### Statistical analysis

#### Observational: mediation

We performed a mediation analysis of the phenotypic traits in males and females separately, using a structural equation modelling framework. Specifically, we used Stata’s *‘sem’* command which fits two regression models: i) a model of the association between the exposure (i.e., BMI/WHR) and any covariates with the mediator (i.e., CRP) (e.g., BMI COVARS—> CRP) and ii) a model of the association between the exposure, mediator and any covariates with the outcome (i.e., grip strength) (e.g., BMI CRP COVARS—> grip strength). From these two models, the direct and total effects are obtained, with the indirect effect calculated using the ‘difference’ method (total effect—direct effect). To ensure comparability across observational and MR analyses, we standardised the adiposity exposures and transformed CRP to the natural log scale (in accordance with the parameterisation of these traits in the respective GWAS). We ran unadjusted and confounder-adjusted models, with the confounders included in the DAG (Supplementary Fig. 1).

#### Genetic: univariable and multivariable MR (MVMR)

We employed a pseudo two-sample design, using genetic association estimates from individual-level data of UKB participants and GWAS summary statistics (described below), to estimate the causal effects of our two adiposity indicators on grip strength and the potential mediating effect of CRP.

(i) Univariable

Univariable MR was performed to identify the total effects of adiposity (i.e., BMI and WHR) on grip strength. The inverse-variance weighted (IVW) method was our main MR model. This method estimates the effect of adiposity on grip strength by averaging the genetic instruments’ ratio of instrument–outcome (SNP-grip strength) to instrument–exposure (SNP-adiposity) association estimates using a multiplicative random effects meta-analysis model^64^. We quantify the extent of heterogeneity between SNP-specific effects by reporting Cochran’s Q-statistic and the I^2^ statistic. SNP-grip strength associations were estimated by sex-specific linear regressions, adjusting for 10 genetic principal components, in UKB. Sex-specific SNP-adiposity associations were extracted from the original GWAS^61, 62^.

 (ii) MVMR

We performed an MVMR (IVW) analysis using SNPs for adiposity and CRP. MVMR is an extension to standard univariable MR, allowing genetic variants to be associated with more than one phenotype and can estimate the direct causal effects of each phenotype in a single analysis^65, 66^. In this way, adiposity (i.e., the exposure) and CRP (i.e., the potential mediator) can be analysed together to quantify the direct effect of adiposity on GS, after accounting for the potential mediating role of CRP^67^. For MVMR analyses, SNPs are included if they achieved genome wide significance with any (but not necessary all) traits under investigation. However, all included SNPs (i.e., those relating to BMI or WHR as well as those relating to CRP) must be independent of each other, so we performed LD clumping (LD r^2^ < 0.001, clumping window > 250 kb). Where a SNP was identified for inclusion but was not available in the other summary data sets (e.g., available in the BMI or WHR GWAS but not in the CRP GWAS (or vice versa)), we selected proxy SNPs with a LD r^2^ > 0.8 and ensured alleles were aligned to the effect-increasing allele (details in supplementary material). Four CRP SNPs were unavailable in the WHR GWAS summary statistics, and no proxies were available. Hence, in the WHR-CRP-grip strength analysis, 84 CRP SNPs were used. Details of the SNPs included in each analysis (including proxies), are provided in Supplementary Tables 2 & 3. As in the univariable MR, SNP-grip strength associations were estimated in UKB, adjusting for 10 genetic principal components. SNP-adiposity and SNP-CRP associations were extracted from the original GWAS.

As recommended for MVMR analyses, we used the conditional F-statistic and generalised versions of Cochran’s Q (Q_A_) to assess instrument strength and heterogeneity in the two-sample setting^68^. To calculate these, the covariance between the effects of the genetic variants on each phenotype was fixed at zero by using non-overlapping samples for each phenotype. As a general rule, the conditional F-statistic should be greater than 10 and Q_A_ estimates should be lower than the relevant critical value, which varies by the number of SNPs but is approximately equal to the number of SNPs included in the model^66, 68^. When the conditional F-statistic or Q_A_ indicated the presence of weak instruments or potential pleiotropy, a Q-minimisation approach (Q_-het_) for estimating robust causal associations was used to supplement the MVMR-IVW approach^68^. This requires a sex-specific correlation matrix between adiposity and CRP, the estimates for which were calculated in the UKB (shown in Supplementary Table 3). For the estimation of confidence intervals using the Q-minimisation approach, 2,000 bootstrap iterations were specified.

To calculate the indirect effect of adiposity on grip strength (via CRP), we used the ‘difference method’ in which the direct effect of adiposity on grip strength from the MVMR analysis is subtracted from the total effect of adiposity on grip strength from the univariable MR analysis^69^, which we describe above. We estimated corresponding standard errors and confidence intervals (CIs) using the ‘propagation of errors’ method^69^.

#### Sensitivity analyses

We performed two univariable MR sensitivity analyses: MR-Egger^70^ and weighted median estimator (MR-WME)^71^ (see details in Supplementary material). We performed further supplementary analyses to explore the validity of our instruments. First, single SNP forest plots were used to summarise the effect of adiposity on grip strength due to each SNP separately; this is helpful for visualising SNP heterogeneity. Second, we tested the sensitivity of our adiposity-grip strength findings to any individual genetic variant, by conducting leave-one-out analyses. Third, we tested associations between adiposity SNPs and potential confounders (listed above), applying a Benjamini–Hochberg false discovery rate of 0.05 to account for multiple testing. Where associations were observed, univariable MR analyses were re-run excluding potentially invalid SNPs. Fourth, funnel plots were used to visually evaluate the direction of pleiotropy, which, if present, would be characterised by asymmetry in the plot. Where pleiotropy was detected visually or by identifying SNPs with a large influence on estimates (determined by Cook’s Distance^72^), univariable MR analyses were re-ran after removing identified SNPs. Similarly, we re-ran the MVMR analyses using an MR-Egger framework^73^. We also re-ran analyses after excluding SNPs i) shown to be associated with potential confounders and ii) exerting a high influence (both of which were determined in the univariable MR analysis described above).

We used Stata16/17, R version 4.2.2 and PLINK1.9/2.0 for data processing and statistical analyses. Observational and univariable MR analyses were performed in Stata^74^, whilst the *MVMR* (version 0.2.0) and *Mendelian Randomization*^75^ (version 0.6.0) R packages were used for the multivariable MR analyses. Observational and genetic results (and sensitivity analyses) are first presented for BMI-GS, followed by WHR-GS.

### Ethics approval

Participants provided informed consent; ethical approval was given by the North-West Multicentre Research Ethics Committee. All methods and data collection procedures were performed in accordance with relevant guidelines and regulations including the Declarations of Helsinki.

### Supplementary Information

Supplementary Information.

## Data Availability

The data that support the findings of this study are available from UK Biobank but restrictions apply to the availability of these data, which were used under license for the current study (application number 71702), and so are not publicly available. Data are however available from the authors upon reasonable request and with permission of UK Biobank. Further details can be found at https://www.ukbiobank.ac.uk.
