# Supplementary material for: Chronic inflammation does not mediate the effect of adiposity on grip strength: results from a multivariable Mendelian randomization study
Source: Sci Rep. 2023 Oct 6;13:16886. doi: 10.1038/s41598-023-43908-y (PMC10558578; doi:10.1038/s41598-023-43908-y)
Supplement: Supplementary file 1 — Supplementary Information. [file 41598_2023_43908_MOESM1_ESM.pdf]

Supplementary material: Does chronic inflammation mediate the effect of adiposity on grip strength? A multivariable Mendelian Randomization study

Norris, T., Sanderson, E., Cooper, R., Garfield, V., Pinto Pereira, S.M.

Participant selection 3

Variable ascertainment 3

Covariates 3

Genotyping, Imputation, and quality control 3

Mendelian randomization (MR) analysis methods and assumptions 4

Univariable MR 4

Inverse-variance weighted 4

MR-Egger regression 4

Weighted median estimator 5

Multivariable MR 5

Allele alignment across GWAS and proxy SNP selection 6

Supplementary Table 1: Summary statistics describing SNP-X associations for the BMI, WHR and CRP instruments: males 8

Supplementary Table 2: Summary statistics describing SNP-X associations for the BMI, WHR and CRP instruments: females 15

Supplementary table 3. Sex-specific phenotypic correlations between adiposity indicators and CRP in UK Biobank participants (n= 367,583) 22

Supplementary table 4. Total, direct and indirect effects of adiposity on GS estimated from structural equation models of observational data (n= 367,583) 23

Supplementary table 5. Estimates from univariable MR for total effect of adiposity* on GS (n= 367,583) 24

Supplementary table 6. Total, direct and indirect effects of 1 SD increase in adiposity trait on GS after accounting for effects of CRP in MVMR (n= 367,583) 25

Supplementary table 7. Estimates from univariable MR for total effect of adiposity* on GS after removing adiposity SNPs associated with potential confounders (n= 367,583) 26

Supplementary table 8. Estimates from univariable MR for total effect of WHR on GS in females after removing three SNPs of high influence (n= 197, 408) 27

Supplementary table 9. Total, direct and indirect effects of 1 SD increase in adiposity trait on GS after removing adiposity SNPs associated with potential confounders (n= 367,583) 28

Supplementary table 10. Direct effect estimates from multivariable MR for total effect of WHR on GS in females after removing three SNPs of high influence (n= 197, 408) 29

Supplementary figure 1. Directed acyclic graph showing tested relationship between adiposity and GS .. 30

Supplementary Figure 2. Funnel plot of risk estimates of BMI SNPs on GS against instrumental strength: Males 31

Supplementary figure 3. Funnel plot of risk estimates of BMI SNPs on GS against instrumental strength: Females 32

Supplementary figure 4. Funnel plot of risk estimates of WHR SNPs on GS against instrumental strength: Males 33

Supplementary figure 5. Funnel plot of risk estimates of WHR SNPs on GS against instrumental strength: Females 34

References 35

Participant selection

At the time of our study, UK Biobank had genetic data available for 487,409 participants. We applied individual-level quality control (QC) to exclude participants with excessive / minimal heterozygosity, sex mismatch, excessive genetic relatedness (more than 10 putative third-degree relatives in the kinship table), no consent, non-European ancestry, and missing QC metrics, leaving 408,480 participants for analysis. We further excluded participants with no phenotype data (N = 40, 897), leaving 367,583 participants for subsequent analyses (**Figure 1**).

Variable ascertainment

Covariates

Potential confounders were identified from a directed acyclic graph constructed using the online tool DAGitty (https://www.dagitty.net). Information on participants' age, sex, smoking status, alcohol intake frequency and physical activity level were collected using a self-reported questionnaire at baseline. Townsend deprivation index was assigned based on postcode as a continuous measure, where a higher index indicates more deprivation. Smoking status was a dichotomous variable defined as not currently smoking vs. currently smoking. Alcohol intake frequency was based on the question *'how often do you drink'*, from which a dichotomous variable was derived representing: alcohol intake less than daily vs. alcohol intake daily/almost daily. Physical activity was based on responses to the question *'Number of days per week of vigorous physical activity lasting at least 10 minutes'*, from which a dichotomous variable was derived: active (at least 4 days/week vigorous physical activity lasting at least 10 minutes) vs. inactive (less than 4 days/week vigorous physical activity lasting at least 10 minutes). Comorbidities (arthritis, asthma and depression) were identified as a binary variable from baseline self-reported illness data using UK Biobank code list.

Genotyping, Imputation, and quality control

Genotyping in UK Biobank was performed using two customised genome-wide arrays, with a genome-wide imputation performed using combined reference panels from UK10K, 1000 Genomes phase 3, and Haplotype Reference Consortium (HRC), resulting in 93,095,623 autosomal variants(1). We further applied quality control to exclude variants with the following criteria: Fisher information <0.3, missing call rate ≥ 5%, or MAF outside of 0.01–1 range.

Mendelian randomization (MR) analysis methods and assumptions

MR analysis uses genetic variants as proxies for exposure of interest to estimate the causal association between the exposure and outcome of interest in an instrumental variable (IV) framework. A conventional MR makes assumptions that genetic instruments for the exposure must be:

- Robustly associated with the exposure. This assumption is reasonable as the genetic variants for each of the traits used in our study achieved genome-wide significance in their respective (large-scale) GWAS.
- Associated with the outcome only through exposure.
- Independent of confounders that influence the exposure and outcome after conditioning on observed confounders(2).

While the last two assumptions cannot be completely verified, we are confident in the validity of our MR estimates because i) we assessed the robustness of our findings to horizontal pleiotropy using two methods (weighted-median regression and MR-Egger) and ii) we checked whether associations existed between our adiposity SNPs and potential (area-level deprivation, smoking status, physical activity, age at recruitment, alcohol intake and comorbidities) using linear or logistic regression as appropriate (we applied a Benjamini-Hochberg false discovery rate (BH-FDR) of 0.05 to account for multiple testing). However, we acknowledge that unobserved confounding may still be present.

Univariable MR

To estimate the total effects of each adiposity indicator on grip strength we performed univariable MR utilising a pseudo two-sample MR approach (where summary statistics for genetic associations with the exposure and the outcome, typically estimated in two independent samples, were used to calculate the MR estimate). Specifically, we used the following methods:

Inverse-variance weighted

For uncorrelated genetic variant j, the causal estimate $\hat{\beta}_{IVW}$ can be calculated by averaging the ratio between variant-outcome association estimate $\hat{\beta}_{Y_j}$ and variant-exposure association estimate $\hat{\beta}_{X_j}$ using the inverse-variance weighted formula for multiplicative random-effect meta-analysis model, which yields:

$$\hat{\beta}_{IVW} = \frac{\sum_j \hat{\beta}_{Y_j} \hat{\beta}_{X_j} \sigma_{Y_j}^{-2}}{\sum_j \hat{\beta}_{X_j}^2 \sigma_{Y_j}^{-2}}$$

where $\hat{\beta}_{Y_j}$ (or $\hat{\beta}_{X_j}$) and σ_{Y_j} are the coefficient and standard error from regression of, e.g., genetic variant j on grip strength Y and genetic variant j on adiposity X(3).

MR-Egger regression

The MR-Egger regression performs a weighted linear regression of $\hat{\beta}_{Y_j}$ on the $\hat{\beta}_{X_j}$, using the $\sigma_{Y_j}^{-2}$ as weights and with unconstrained intercept[11]. The causal estimate $\hat{\beta}_E$ is obtained from the model:

$$\hat{\beta}_{Y_j} = \hat{\alpha}_E + \hat{\beta}_E \hat{\beta}_{X_j}$$

The intercept term $\hat{\alpha}_E$ denotes the estimated horizontal pleiotropic effect across genetic variants, and thus, the associated p-value for the intercept term is indicative of overall horizontal pleiotropy. The MR-Egger estimates typically have low power, but we used this method to detect horizontal pleiotropy(4).

Both the IVW and MR-Egger models make several additional assumptions, including NO Measurement Error (NOME), InSIDE (Instrument Strength Independent of Direct Effect), and VIS (Variation in Instrument Strength), as detailed elsewhere(5).

Weighted median estimator

In the weighted median estimator, first the causal estimate $\hat{\beta}$ of each variant j is calculated with the ratio method as $\hat{\beta}_j = \hat{\beta}_{Y_j}/\hat{\beta}_{X_j}$. Then, $\hat{\beta}_j$ are sorted (so that $\hat{\beta}_1 < \hat{\beta}_2 < \cdots < \hat{\beta}_J$) and standardised weight w_j is assigned to the jth-ordered ratio estimate. The weights are calculated using the inverse variance of the ratio estimates as $\acute{w}_j = \hat{\beta}_{X_j}\sigma_{Y_j}^{-2}$. The standardised weights are $w_j = \acute{w}_j/\sum_j \acute{w}_j$ and their sum is $s_j = 1$.

If k denotes the largest integer such that the sum of weights up to and including the kth estimate ($s_k = \sum_{j \le k} w_j$) is <0.5, the causal estimate from weighted median method $\hat{\beta}_{WME}$ can be calculated by interpolation between the kth and $(k+1)$th ratio estimates as follows:

$$\hat{\beta}_{WME} = \hat{\beta}_k + \left(\hat{\beta}_{k+1} - \hat{\beta}_k\right) \times \frac{0.5 - s_k}{s_{k+1} - s_k}$$

This approach should provide a consistent estimate given that at least 50% of the weights are derived from valid variants and is more robust to violation of the untestable InSIDE assumption(6, 7).

Multivariable MR

MVMR requires a set of SNPs which are associated with the exposure/mediator variables but do not affect the outcome other than through these variables. As per the standard univariable MR with a single exposure, these SNPs are used to predict each of the exposure variables in the model and these predicted values are then used to estimate the effect of the exposures on the outcome in a multivariable regression analysis.

To perform an MVMR analysis, the number of included instruments must but be at least equal to the number of exposures to be instrumented in the model. As with standard univariable MR it is important that the instrumental variable assumptions are satisfied. In MVMR these assumptions become:

- MVMR-IV1: the exposures/mediators must be strongly predicted by the SNPs given the other exposures/mediators included in the model;
- MVMR-IV2: the SNPs must be independent of the outcome given all of the exposures/mediators included in the model; and
- MVMR- IV3: the SNPs must be independent of all confounders of any of the exposures/mediators and the outcome Y.

MVMR-IV1: In MVMR it is necessary for the exposures/mediators to be strongly predicted by the set of SNPs; the exposures/mediators must also be strongly predicted by the SNPs conditional on the other exposures/mediators included in the estimation. In our example in which adiposity and CRP are included as exposures/mediators, the set of SNPs used as instruments must be able to strongly predict, e.g., adiposity, once the association between the SNPs and CRP has been accounted for(8, 9). If this assumption is not satisfied then there will be multicollinearity between the predicted exposures/mediators and the MVMR estimates obtained will suffer from weak-instrument bias(8, 10).

MVMR-IV2/MVMR-IV3: MVMR analysis requires that the SNPs do not affect the outcome other than through the set of exposures/mediators included in the model. However, SNPs that affect multiple phenotypes can be included in the estimation, as long as all of those phenotypes are included as exposures/mediators. These assumptions are therefore slightly less restrictive than for univariable MR. Pleiotropy, specifically horizontal pleiotropy in which a SNP is associated with two or more phenotypes independently, is a violation of the traditional third IV assumption if any phenotype associated with the SNP (other than the exposure of interest) is also associated with the outcome. MVMR can be used as a form of pleiotropy adjustment when SNPs have potential horizontal pleiotropic effects on a phenotype that influences the outcome as this phenotype is included as an additional exposure in the analysis, therefore accounting for the pleiotropic pathway. It is still necessary however to test for heterogeneity in the SNP-outcome association as an indicator of potential pleiotropy in MVMR that has not been accounted for and this can be achieved using an adjusted Cochran Q statistic with summary data(9). There are currently few methods for sensitivity analysis in MVMR if pleiotropy is suspected, however one method of analysis that can be used to adjust for pleiotropy is multivariable MR-Egger(11) which allows for directional pleiotropy which is uncorrelated with the size of the SNP-exposure association.

Allele alignment across GWAS and proxy SNP selection

In MVMR analyses, the effect of each SNP has to be obtained for its associated trait and for the other trait in the analysis (e.g., beta estimates for the 76 BMI SNPs have to be obtained for the effect on both BMI and CRP). When identifying the SNPs in the GWAS of the other trait, we ensured that the beta from the same allele selected as the ‘effect increasing’ allele in the first GWAS (e.g., BMI) was selected from the other GWAS (e.g., CRP). If there was a mismatch between the alleles across the two GWAS, then the beta in the second GWAS was multiplied by -1 to ensure alignment.

Where SNPs selected from the exposure GWAS (e.g., BMI or WHR) were not available in the mediator GWAS (CRP), or vice versa, proxy SNPs were identified using the ‘proxy search’ facility on SNIPA (https://snipa.helmholtz-muenchen.de/snipa3/), with those selected having an LD R^2>0.8. Where proxy SNPs were used, we checked which allele in the proxy SNP matched which allele in the original SNP. The allele frequency was used to check if this was the effect allele in the GWAS. If the allele frequency matches that of the effect allele in the GWAS then transformation of the beta was not required. If the effect allele frequency in the GWAS was for the other allele then we multiplied the reported beta by -1 to get the beta for us to use. For example, SNP rs2287019 from the BMI GWAS was not observed in the CRP GWAS and so we selected the proxy rs11672660. In the BMI GWAS, allele C (allele frequency=0.803) was used as the effect increasing allele whereas

in the CRP GWAS, allele T (allele frequency=0.214) was reported as the effect allele for the proxy SNP. As the allele frequencies suggest that different alleles were used across the two GWAS, we multiplied the beta for the effect of rs11672660 on CRP by -1 to ensure alignment of alleles across the two GWAS.

Supplementary Table 1: Summary statistics describing SNP-X associations for the BMI, WHR and CRP instruments: males

Instrument	SNP ID	Chr	Effect allele	Other allele	Beta*	SE	*p-value*	*Included in CRP GWS*	*Proxy SNP*[†]	*LD R^2*
BMI	rs1000940	17	G	A	0.0184	0.0045	1.28E-08	Yes	N/A	N/A
BMI	rs10132280	14	C	A	0.0289	0.0044	1.14E-11	Yes	N/A	N/A
BMI	rs1016287	2	T	C	0.0204	0.0044	2.25E-11	No	rs887912	0.99
BMI	rs10182181	2	G	A	0.0234	0.004	8.78E-24	Yes	N/A	N/A
BMI	rs10733682	9	A	G	0.0114	0.0041	1.83E-08	Yes	N/A	N/A
BMI	rs10938397	4	G	A	0.0402	0.0041	3.20E-38	Yes	N/A	N/A
BMI	rs10968576	9	G	A	0.0197	0.0043	6.61E-14	Yes	N/A	N/A
BMI	rs11030104	11	A	G	0.0445	0.005	5.56E-28	Yes	N/A	N/A
BMI	rs11057405	12	G	A	0.027	0.0072	2.02E-08	Yes	N/A	N/A
BMI	rs11126666	2	A	G	0.0258	0.0045	1.33E-09	Yes	N/A	N/A
BMI	rs11165643	1	T	C	0.0207	0.0041	2.07E-12	Yes	N/A	N/A
BMI	rs11191560	10	C	T	0.0313	0.0071	8.45E-09	Yes	N/A	N/A
BMI	rs11583200	1	C	T	0.0174	0.0042	1.48E-08	Yes	N/A	N/A
BMI	rs1167827	7	G	A	0.0234	0.0044	6.33E-10	No	rs17207196	0.91
BMI	rs11688816	2	G	A	0.0145	0.0041	1.89E-08	Yes	N/A	N/A
BMI	rs11727676	4	T	C	0.0403	0.0084	2.55E-08	Yes	N/A	N/A
BMI	rs11847697	14	T	C	0.0452	0.011	3.99E-09	Yes	N/A	N/A
BMI	rs12286929	11	G	A	0.0222	0.004	1.31E-12	Yes	N/A	N/A
BMI	rs12401738	1	A	G	0.016	0.0043	1.15E-10	Yes	N/A	N/A
BMI	rs12429545	13	A	G	0.0348	0.0062	1.09E-12	Yes	N/A	N/A
BMI	rs12446632	16	G	A	0.0385	0.006	1.48E-18	Yes	N/A	N/A
BMI	rs12566985	1	G	A	0.0216	0.0041	3.28E-15	Yes	N/A	N/A
BMI	rs12885454	14	C	A	0.0188	0.0042	1.94E-10	Yes	N/A	N/A
BMI	rs12940622	17	G	A	0.0206	0.0041	2.49E-09	Yes	N/A	N/A
BMI	rs13021737	2	G	A	0.052	0.0054	1.11E-50	Yes	N/A	N/A
BMI	rs13078960	3	G	T	0.0258	0.0051	1.74E-14	Yes	N/A	N/A

BMI	rs13107325	4	T	C	0.0525	0.0088	1.82E-12	Yes	N/A	N/A
BMI	rs13191362	6	A	G	0.0245	0.0063	7.34E-09	Yes	N/A	N/A
BMI	rs1516725	3	C	T	0.0439	0.0062	1.89E-22	Yes	N/A	N/A
BMI	rs1528435	2	T	C	0.0229	0.0042	1.20E-08	Yes	N/A	N/A
BMI	rs1558902	16	A	T	0.0842	0.0041	7.50E-153	Yes	N/A	N/A
BMI	rs16851483	3	T	G	0.0433	0.0103	3.55E-10	Yes	N/A	N/A
BMI	rs16951275	15	T	C	0.032	0.0049	1.91E-17	Yes	N/A	N/A
BMI	rs17001654	4	G	C	0.0278	0.0072	7.76E-09	Yes	N/A	N/A
BMI	rs17024393	1	C	T	0.0605	0.0116	7.03E-14	Yes	N/A	N/A
BMI	rs17094222	10	C	T	0.0307	0.005	5.94E-11	Yes	N/A	N/A
BMI	rs17405819	8	T	C	0.0208	0.0044	2.07E-11	Yes	N/A	N/A
BMI	rs17724992	19	A	G	0.0228	0.0047	3.41E-08	Yes	N/A	N/A
BMI	rs1808579	18	C	T	0.0104	0.0041	4.17E-08	Yes	N/A	N/A
BMI	rs1928295	9	T	C	0.0119	0.004	7.91E-10	Yes	N/A	N/A
BMI	rs2033529	6	G	A	0.018	0.0044	1.39E-08	Yes	N/A	N/A
BMI	rs2033732	8	C	T	0.0187	0.0047	4.89E-08	Yes	N/A	N/A
BMI	rs205262	6	G	A	0.0166	0.0046	1.75E-10	Yes	N/A	N/A
BMI	rs2075650	19	A	G	0.0206	0.006	1.25E-08	Yes	N/A	N/A
BMI	rs2112347	5	T	G	0.0209	0.0042	6.19E-17	Yes	N/A	N/A
BMI	rs2121279	2	T	C	0.029	0.0057	2.31E-08	Yes	N/A	N/A
BMI	rs2176598	11	T	C	0.0237	0.0047	2.97E-08	Yes	N/A	N/A
BMI	rs2207139	6	G	A	0.044	0.0053	4.13E-29	Yes	N/A	N/A
BMI	rs2245368	7	C	T	0.0344	0.0071	3.19E-08	Yes	N/A	N/A
BMI	rs2287019	19	C	T	0.0386	0.0055	4.58E-18	No	rs11672660	0.89
BMI	rs2365389	3	C	T	0.0193	0.0041	1.63E-10	Yes	N/A	N/A
BMI	rs2650492	16	A	G	0.0213	0.0047	1.92E-09	Yes	N/A	N/A
BMI	rs2820292	1	C	A	0.0225	0.004	1.83E-10	Yes	N/A	N/A
BMI	rs29941	19	G	A	0.0176	0.0042	2.41E-08	Yes	N/A	N/A
BMI	rs3101336	1	C	T	0.0346	0.0042	2.66E-26	Yes	N/A	N/A
BMI	rs3736485	15	A	G	0.0167	0.0041	7.41E-09	Yes	N/A	N/A

BMI	rs3810291	19	A	G	0.0277	0.0048	4.81E-15	Yes	N/A	N/A
BMI	rs3817334	11	T	C	0.0263	0.0041	5.15E-17	Yes	N/A	N/A
BMI	rs3849570	3	A	C	0.0233	0.0045	2.60E-08	No	rs11710970	0.91
BMI	rs3888190	16	A	C	0.035	0.0041	3.14E-23	Yes	N/A	N/A
BMI	rs4256980	11	G	C	0.0186	0.0042	2.90E-11	Yes	N/A	N/A
BMI	rs4740619	9	T	C	0.0198	0.004	4.56E-09	Yes	N/A	N/A
BMI	rs543874	1	G	A	0.0341	0.0051	2.62E-35	Yes	N/A	N/A
BMI	rs6477694	9	C	T	0.0136	0.0042	2.67E-08	Yes	N/A	N/A
BMI	rs6567160	18	C	T	0.0546	0.0048	3.93E-53	Yes	N/A	N/A
BMI	rs657452	1	A	G	0.0216	0.0042	5.48E-13	Yes	N/A	N/A
BMI	rs6804842	3	G	A	0.0202	0.0041	2.48E-09	Yes	N/A	N/A
BMI	rs7138803	12	A	G	0.0273	0.0042	8.15E-24	Yes	N/A	N/A
BMI	rs7141420	14	T	C	0.0218	0.004	1.23E-14	Yes	N/A	N/A
BMI	rs7243357	18	T	G	0.0254	0.0053	3.86E-08	Yes	N/A	N/A
BMI	rs758747	16	T	C	0.0259	0.0048	7.47E-10	Yes	N/A	N/A
BMI	rs7599312	2	G	A	0.0256	0.0045	1.17E-10	Yes	N/A	N/A
BMI	rs7899106	10	G	A	0.036	0.0094	2.96E-08	Yes	N/A	N/A
BMI	rs7903146	10	C	T	0.0294	0.0045	1.11E-11	Yes	N/A	N/A
BMI	rs9400239	6	C	T	0.015	0.0043	1.61E-08	Yes	N/A	N/A
BMI	rs9925964	16	A	G	0.0184	0.0042	8.11E-10	Yes	N/A	N/A
WHR	rs1011731	1	G	A	0.014	0.005	1.07E-08	Yes	N/A	N/A
WHR	rs10195252	2	C	T	0.009	0.005	2.57E-09	Yes	N/A	N/A
WHR	rs10245353	7	A	C	0.019	0.006	1.57E-10	Yes	N/A	N/A
WHR	rs11048470	12	T	G	0.016	0.005	6.33E-12	Yes	N/A	N/A
WHR	rs1121980	16	A	G	0.048	0.005	1.33E-38	Yes	N/A	N/A
WHR	rs11663816	18	C	T	0.019	0.006	2.65E-11	Yes	N/A	N/A
WHR	rs12549058	8	G	T	0.033	0.009	3.17E-10	No	rs16937647	0.92
WHR	rs1294421	6	G	T	0.018	0.005	6.93E-14	Yes	N/A	N/A
WHR	rs1358980	6	T	C	0.003	0.005	1.98E-14	Yes	N/A	N/A
WHR	rs1440372	15	C	T	0.021	0.005	7.59E-09	Yes	N/A	N/A

WHR	rs1443512	12	A	C	0.012	0.006	2.76E-11		Yes		N/A		N/A
WHR	rs1569135	2	A	G	0.026	0.005	1.00E-12		Yes		N/A		N/A
WHR	rs16996700	20	T	C	0.021	0.005	1.60E-08		Yes		N/A		N/A
WHR	rs17109256	14	A	G	0.029	0.006	3.022E-08		Yes		N/A		N/A
WHR	rs17451107	3	T	C	0.026	0.005	3.50E-11		Yes		N/A		N/A
WHR	rs2075650	19	A	G	0.026	0.007	6.43E-09		Yes		N/A		N/A
WHR	rs2179129	22	A	G	0.016	0.005	1.24E-09		Yes		N/A		N/A
WHR	rs2287019	19	C	T	0.034	0.007	4.344E-09		No		rs11672660		0.89
WHR	rs2765539	1	T	C	0.022	0.006	1.08E-12		Yes		N/A		N/A
WHR	rs3786897	19	G	A	0.025	0.005	3.95E-11		Yes		N/A		N/A
WHR	rs459193	5	A	G	0.026	0.005	6.02E-12		Yes		N/A		N/A
WHR	rs4640244	17	G	A	0.017	0.005	3.11E-08		Yes		N/A		N/A
WHR	rs4846565	1	A	G	0.004	0.005	4.75E-11		Yes		N/A		N/A
WHR	rs4929927	11	G	A	0.018	0.005	7.60E-09		Yes		N/A		N/A
WHR	rs7801581	7	T	C	0.024	0.006	4.951E-08		Yes		N/A		N/A
WHR	rs929641	2	A	G	0.018	0.005	4.25E-09		Yes		N/A		N/A
WHR	rs9491696	6	G	C	0.03	0.005	4.88E-30		Yes		N/A		N/A
WHR	rs9860730	3	G	A	0.0004	0.005	2.84E-10		Yes		N/A		N/A
**Instrument**	**SNP ID**	**Chr**	**Effect allele**	**Other allele**	**Beta***	**SE**	***p-value***	***Included in adiposity GWAS***		***Proxy SNP***		***LD R^2***	
								BMI GWAS	***WHR GWAS***	***BMI GWAS***	***WHR GWAS***	***BMI GWAS***	***WHR GWAS***
CRP	rs10089518	8	C	T	0.025746	0.003807	1.39E-11	Yes	Yes	N/A	N/A	N/A	N/A
CRP	rs10240168	7	C	G	0.028684	0.004343	4.11E-11	Yes	Yes	N/A	N/A	N/A	N/A
CRP	rs10512597	17	C	T	0.036931	0.00489	4.44E-14	Yes	Yes	N/A	N/A	N/A	N/A
CRP	rs1051338	10	G	T	0.023881	0.003992	2.27E-09	Yes	Yes	N/A	N/A	N/A	N/A
CRP	rs10521222	16	C	T	0.104411	0.010714	2.06E-22	Yes	No	N/A	None available	N/A	N/A
CRP	rs10778215	12	T	A	0.033242	0.003583	1.86E-20	Yes	Yes	N/A	N/A	N/A	N/A
CRP	rs10832027	11	A	G	0.025944	0.003745	4.43E-12	Yes	Yes	N/A	N/A	N/A	N/A

CRP	rs10925027	1	T	C	0.036035	0.00382	4.25E-21	Yes	Yes	N/A	N/A	N/A	N/A
CRP	rs11108056	12	C	G	0.027907	0.003708	5.42E-14	Yes	Yes	N/A	N/A	N/A	N/A
CRP	rs11615578	12	T	C	0.047139	0.004401	9.80E-27	Yes	Yes	N/A	N/A	N/A	N/A
CRP	rs11783705	8	C	A	0.032575	0.00428	2.85E-14	Yes	Yes	N/A	N/A	N/A	N/A
CRP	rs12043437	1	T	C	0.04123	0.005901	2.91E-12	Yes	Yes	N/A	N/A	N/A	N/A
CRP	rs12202641	6	C	T	0.022804	0.003617	3.00E-10	Yes	Yes	N/A	N/A	N/A	N/A
CRP	rs12310160	12	C	T	0.051711	0.005215	3.82E-23	Yes	Yes	N/A	N/A	N/A	N/A
CRP	rs12587622	14	G	A	0.020798	0.003609	8.52E-09	Yes	Yes	N/A	N/A	N/A	N/A
CRP	rs1260326	2	T	C	0.073462	0.003604	2.72E-92	Yes	Yes	N/A	N/A	N/A	N/A
CRP	rs12960928	18	C	T	0.024	0.003993	1.91E-09	Yes	Yes	N/A	N/A	N/A	N/A
CRP	rs12995480	2	C	T	0.031261	0.004855	1.24E-10	Yes	Yes	N/A	N/A	N/A	N/A
CRP	rs13030345	2	T	G	0.052982	0.004989	2.60E-26	Yes	Yes	N/A	N/A	N/A	N/A
CRP	rs13233571	7	C	T	0.056895	0.005476	2.95E-25	Yes	Yes	N/A	N/A	N/A	N/A
CRP	rs13282752	8	A	G	0.036001	0.004433	4.85E-16	Yes	Yes	N/A	N/A	N/A	N/A
CRP	rs13409371	2	A	G	0.048232	0.003847	5.07E-36	Yes	Yes	N/A	N/A	N/A	N/A
CRP	rs1371614	2	C	T	0.026321	0.004234	5.23E-10	Yes	Yes	N/A	N/A	N/A	N/A
CRP	rs1441169	2	A	G	0.024926	0.003725	2.27E-11	Yes	Yes	N/A	N/A	N/A	N/A
CRP	rs1490384	6	C	T	0.024816	0.003545	2.65E-12	Yes	Yes	N/A	N/A	N/A	N/A
CRP	rs1509394	2	T	C	0.025685	0.004147	6.05E-10	Yes	Yes	N/A	N/A	N/A	N/A
CRP	rs1558902	16	A	T	0.033926	0.003701	5.20E-20	Yes	Yes	N/A	N/A	N/A	N/A
CRP	rs1582763	11	G	A	0.022107	0.0037	2.37E-09	Yes	Yes	N/A	N/A	N/A	N/A
CRP	rs1616534	8	T	C	0.030177	0.003703	3.88E-16	Yes	Yes	N/A	N/A	N/A	N/A
CRP	rs1659685	2	T	C	0.026139	0.003886	1.80E-11	Yes	Yes	N/A	N/A	N/A	N/A
CRP	rs17149760	8	A	G	0.038914	0.004292	1.31E-19	Yes	Yes	N/A	N/A	N/A	N/A
CRP	rs17616063	16	A	G	0.106356	0.011169	1.80E-21	Yes	No	N/A	None available	N/A	N/A
CRP	rs17643262	19	G	A	0.033345	0.005874	1.41E-08	Yes	Yes	N/A	N/A	N/A	N/A
CRP	rs17658229	5	C	T	0.055568	0.009522	5.50E-09	Yes	Yes	N/A	N/A	N/A	N/A
CRP	rs17759740	2	T	A	0.033895	0.005937	1.16E-08	Yes	Yes	N/A	N/A	N/A	N/A
CRP	rs178810	17	T	C	0.02001	0.003606	2.95E-08	Yes	Yes	N/A	N/A	N/A	N/A

CRP	rs1800961	20	C	T	0.1115	0.011267	4.63E-23	Yes	Yes	N/A	N/A	N/A	N/A
CRP	rs1805096	1	G	A	0.104381	0.003614	2.17E-183	Yes	Yes	N/A	N/A	N/A	N/A
CRP	rs1880241	7	A	G	0.027537	0.003687	8.41E-14	Yes	Yes	N/A	N/A	N/A	N/A
CRP	rs1985096	19	A	T	0.042135	0.006467	7.48E-11	Yes	Yes	N/A	N/A	N/A	N/A
CRP	rs2064009	8	T	C	0.027111	0.003549	2.28E-14	Yes	Yes	N/A	N/A	N/A	N/A
CRP	rs206967	12	C	T	0.027577	0.004797	9.20E-09	Yes	Yes	N/A	N/A	N/A	N/A
CRP	rs2239222	14	G	A	0.035484	0.003901	9.87E-20	Yes	Yes	N/A	N/A	N/A	N/A
CRP	rs2293476	1	C	G	0.030262	0.004225	8.27E-13	Yes	Yes	N/A	N/A	N/A	N/A
CRP	rs2315008	20	G	T	0.023467	0.003777	5.36E-10	Yes	Yes	N/A	N/A	N/A	N/A
CRP	rs2352975	3	C	T	0.024897	0.004026	6.43E-10	Yes	Yes	N/A	N/A	N/A	N/A
CRP	rs2572397	8	A	G	0.025043	0.003635	5.85E-12	Yes	Yes	N/A	N/A	N/A	N/A
CRP	rs2686343	12	A	G	0.023413	0.003749	4.35E-10	Yes	Yes	N/A	N/A	N/A	N/A
CRP	rs2710804	7	C	T	0.021262	0.003737	1.30E-08	Yes	Yes	N/A	N/A	N/A	N/A
CRP	rs2794520	1	C	T	0.182186	0.003712	1.02E-305	Yes	Yes	N/A	N/A	N/A	N/A
CRP	rs2798604	1	T	G	0.021868	0.003837	1.24E-08	Yes	Yes	N/A	N/A	N/A	N/A
CRP	rs2801578	1	T	A	0.04819	0.003913	8.09E-35	Yes	Yes	N/A	N/A	N/A	N/A
CRP	rs2836878	21	G	A	0.042902	0.004079	7.71E-26	Yes	Yes	N/A	N/A	N/A	N/A
CRP	rs2852151	18	A	G	0.024735	0.003655	1.36E-11	Yes	Yes	N/A	N/A	N/A	N/A
CRP	rs2891677	8	T	C	0.019859	0.003511	1.59E-08	Yes	Yes	N/A	N/A	N/A	N/A
CRP	rs2948305	8	A	G	0.023948	0.003778	2.39E-10	Yes	Yes	N/A	N/A	N/A	N/A
CRP	rs2965109	19	T	C	0.025284	0.00414	1.04E-09	Yes	Yes	N/A	N/A	N/A	N/A
CRP	rs3134899	6	T	C	0.023329	0.004274	4.93E-08	Yes	Yes	N/A	N/A	N/A	N/A
CRP	rs340005	15	A	G	0.030007	0.003736	1.01E-15	Yes	Yes	N/A	N/A	N/A	N/A
CRP	rs4075359	8	T	C	0.026545	0.003705	8.07E-13	Yes	Yes	N/A	N/A	N/A	N/A
CRP	rs4092465	18	G	A	0.027483	0.004364	3.11E-10	Yes	Yes	N/A	N/A	N/A	N/A
CRP	rs4129267	1	C	T	0.087519	0.003612	1.20E-129	Yes	Yes	N/A	N/A	N/A	N/A
CRP	rs4147730	11	G	A	0.027431	0.005016	4.65E-08	Yes	Yes	N/A	N/A	N/A	N/A
CRP	rs4246598	2	A	C	0.022063	0.003547	5.11E-10	Yes	Yes	N/A	N/A	N/A	N/A
CRP	rs4420638	19	A	G	0.229459	0.006122	2.26E-307	Yes	No	N/A	None available	N/A	N/A

CRP	rs4660443	1	T	C	0.028822	0.004684	7.80E-10	Yes	Yes	N/A	N/A	N/A	N/A
CRP	rs469772	1	C	T	0.031327	0.004542	5.54E-12	Yes	Yes	N/A	N/A	N/A	N/A
CRP	rs4767878	12	G	A	0.087865	0.01311	2.13E-11	Yes	No	N/A	None available	N/A	N/A
CRP	rs4774590	15	G	A	0.022169	0.003985	2.71E-08	Yes	Yes	N/A	N/A	N/A	N/A
CRP	rs4841132	8	G	A	0.065095	0.006243	2.00E-25	Yes	Yes	N/A	N/A	N/A	N/A
CRP	rs4841407	8	G	A	0.025494	0.003677	4.24E-12	Yes	Yes	N/A	N/A	N/A	N/A
CRP	rs541619	12	A	G	0.075692	0.004812	1.04E-55	Yes	Yes	N/A	N/A	N/A	N/A
CRP	rs6001193	22	A	G	0.027809	0.003706	6.53E-14	Yes	Yes	N/A	N/A	N/A	N/A
CRP	rs644234	9	G	T	0.022597	0.003708	1.13E-09	Yes	Yes	N/A	N/A	N/A	N/A
CRP	rs6485716	11	G	A	0.028949	0.005029	8.81E-09	Yes	Yes	N/A	N/A	N/A	N/A
CRP	rs6485751	11	G	C	0.03089	0.004359	1.43E-12	Yes	Yes	N/A	N/A	N/A	N/A
CRP	rs660895	6	G	A	0.034428	0.004525	2.89E-14	Yes	Yes	N/A	N/A	N/A	N/A
CRP	rs6672992	1	C	A	0.050172	0.005563	2.00E-19	Yes	Yes	N/A	N/A	N/A	N/A
CRP	rs680925	1	A	G	0.030454	0.003734	3.64E-16	Yes	Yes	N/A	N/A	N/A	N/A
CRP	rs7015461	8	T	C	0.028417	0.004287	3.50E-11	Yes	Yes	N/A	N/A	N/A	N/A
CRP	rs707939	6	A	C	0.024086	0.003858	4.44E-10	Yes	Yes	N/A	N/A	N/A	N/A
CRP	rs7310409	12	G	A	0.137075	0.003706	2.54E-299	Yes	Yes	N/A	N/A	N/A	N/A
CRP	rs7837587	8	C	T	0.026823	0.003537	3.53E-14	Yes	Yes	N/A	N/A	N/A	N/A
CRP	rs863018	1	C	T	0.043948	0.003941	7.56E-29	Yes	Yes	N/A	N/A	N/A	N/A
CRP	rs9284725	2	C	A	0.02731	0.00419	7.34E-11	Yes	Yes	N/A	N/A	N/A	N/A
CRP	rs937813	2	C	T	0.047625	0.006223	2.05E-14	Yes	Yes	N/A	N/A	N/A	N/A
CRP	rs9385532	6	C	T	0.02556	0.003804	1.90E-11	Yes	Yes	N/A	N/A	N/A	N/A
CRP	rs9929889	16	T	C	0.02528	0.004085	6.24E-10	Yes	Yes	N/A	N/A	N/A	N/A

*****Where necessary, beta coefficients were multiplied by -1 to ensure all betas represented an increase in the respective traits and allele harmonisation was done to ensure alignment of alleles for both the SNP-X and SNP-Y associations. GWAS betas, SEs and p-values taken from Locke et al. 2015. Nature, 518(7538):197-206 (BMI); Shungin et al. 2015. Nature, 518(7538):187-96 (WHR); Ligthart et al. 2018. AJHG, 103(5): 691-706 (CRP); [†]proxy SNPs identified using online tool ‘SNIPA’ https://snipa.helmholtz-muenchen.de/snipa3/

Supplementary Table 2: Summary statistics describing SNP-X associations for the BMI, WHR and CRP instruments: females

Instrument	SNP ID	Chr	Effect allele	Other allele	Beta*	SE	*p-value*	*Included in CRP GWAS*	*Proxy SNP*[†]	*LD R^2*
BMI	rs1000940	17	G	A	0.0203	0.0043	1.28E-08	Yes	N/A	N/A
BMI	rs10132280	14	C	A	0.0171	0.0043	1.14E-11	Yes	N/A	N/A
BMI	rs1016287	2	T	C	0.0254	0.0044	2.25E-11	No	rs887912	0.99
BMI	rs10182181	2	G	A	0.0366	0.0039	8.78E-24	Yes	N/A	N/A
BMI	rs10733682	9	A	G	0.0229	0.0041	1.83E-08	Yes	N/A	N/A
BMI	rs10938397	4	G	A	0.0404	0.0041	3.20E-38	Yes	N/A	N/A
BMI	rs10968576	9	G	A	0.0289	0.0043	6.61E-14	Yes	N/A	N/A
BMI	rs11030104	11	A	G	0.0384	0.0048	5.56E-28	Yes	N/A	N/A
BMI	rs11057405	12	G	A	0.0334	0.007	2.02E-08	Yes	N/A	N/A
BMI	rs11126666	2	A	G	0.016	0.0044	1.33E-09	Yes	N/A	N/A
BMI	rs11165643	1	T	C	0.023	0.004	2.07E-12	Yes	N/A	N/A
BMI	rs11191560	10	C	T	0.0307	0.0069	8.45E-09	Yes	N/A	N/A
BMI	rs11583200	1	C	T	0.0175	0.0041	1.48E-08	Yes	N/A	N/A
BMI	rs1167827	7	G	A	0.0172	0.0042	6.33E-10	No	rs17207196	0.91
BMI	rs11688816	2	G	A	0.019	0.0039	1.89E-08	Yes	N/A	N/A
BMI	rs11727676	4	T	C	0.0312	0.0083	2.55E-08	Yes	N/A	N/A
BMI	rs11847697	14	T	C	0.0532	0.0106	3.99E-09	Yes	N/A	N/A
BMI	rs12286929	11	G	A	0.0207	0.0039	1.31E-12	Yes	N/A	N/A
BMI	rs12401738	1	A	G	0.0256	0.0041	1.15E-10	Yes	N/A	N/A
BMI	rs12429545	13	A	G	0.0322	0.006	1.09E-12	Yes	N/A	N/A
BMI	rs12446632	16	G	A	0.0432	0.0058	1.48E-18	Yes	N/A	N/A
BMI	rs12566985	1	G	A	0.027	0.004	3.28E-15	Yes	N/A	N/A
BMI	rs12885454	14	C	A	0.0228	0.0041	1.94E-10	Yes	N/A	N/A
BMI	rs12940622	17	G	A	0.0161	0.004	2.49E-09	Yes	N/A	N/A
BMI	rs13021737	2	G	A	0.0686	0.0052	1.11E-50	Yes	N/A	N/A
BMI	rs13078960	3	G	T	0.0336	0.005	1.74E-14	Yes	N/A	N/A

BMI	rs13107325	4	T	C	0.0446	0.0087	1.82E-12	Yes	N/A	N/A
BMI	rs13191362	6	A	G	0.03	0.0062	7.34E-09	Yes	N/A	N/A
BMI	rs1516725	3	C	T	0.0466	0.0059	1.89E-22	Yes	N/A	N/A
BMI	rs1528435	2	T	C	0.0137	0.0041	1.20E-08	Yes	N/A	N/A
BMI	rs1558902	16	A	T	0.0791	0.0041	7.50E-153	Yes	N/A	N/A
BMI	rs16851483	3	T	G	0.0524	0.0096	3.55E-10	Yes	N/A	N/A
BMI	rs16951275	15	T	C	0.0302	0.0048	1.91E-17	Yes	N/A	N/A
BMI	rs17001654	4	G	C	0.0322	0.0067	7.76E-09	Yes	N/A	N/A
BMI	rs17024393	1	C	T	0.0713	0.0114	7.03E-14	Yes	N/A	N/A
BMI	rs17094222	10	C	T	0.0204	0.0049	5.94E-11	Yes	N/A	N/A
BMI	rs17405819	8	T	C	0.0243	0.0043	2.07E-11	Yes	N/A	N/A
BMI	rs17724992	19	A	G	0.0159	0.0045	3.41E-08	Yes	N/A	N/A
BMI	rs1808579	18	C	T	0.0224	0.0039	4.17E-08	Yes	N/A	N/A
BMI	rs1928295	9	T	C	0.0258	0.0039	7.91E-10	Yes	N/A	N/A
BMI	rs2033529	6	G	A	0.0203	0.0043	1.39E-08	Yes	N/A	N/A
BMI	rs2033732	8	C	T	0.0193	0.0045	4.89E-08	Yes	N/A	N/A
BMI	rs205262	6	G	A	0.0268	0.0045	1.75E-10	Yes	N/A	N/A
BMI	rs2075650	19	A	G	0.031	0.0058	1.25E-08	Yes	N/A	N/A
BMI	rs2112347	5	T	G	0.0298	0.0041	6.19E-17	Yes	N/A	N/A
BMI	rs2121279	2	T	C	0.021	0.0056	2.31E-08	Yes	N/A	N/A
BMI	rs2176598	11	T	C	0.0167	0.0045	2.97E-08	Yes	N/A	N/A
BMI	rs2207139	6	G	A	0.0465	0.0052	4.13E-29	Yes	N/A	N/A
BMI	rs2245368	7	C	T	0.0281	0.0078	3.19E-08	Yes	N/A	N/A
BMI	rs2287019	19	C	T	0.0324	0.0053	4.58E-18	No	rs11672660	0.89
BMI	rs2365389	3	C	T	0.0203	0.004	1.63E-10	Yes	N/A	N/A
BMI	rs2650492	16	A	G	0.02	0.0044	1.92E-09	Yes	N/A	N/A
BMI	rs2820292	1	C	A	0.018	0.0039	1.83E-10	Yes	N/A	N/A
BMI	rs29941	19	G	A	0.0187	0.0042	2.41E-08	Yes	N/A	N/A
BMI	rs3101336	1	C	T	0.0334	0.004	2.66E-26	Yes	N/A	N/A
BMI	rs3736485	15	A	G	0.0185	0.0039	7.41E-09	Yes	N/A	N/A

BMI	rs3810291	19	A	G	0.0295	0.0047	4.81E-15	Yes	N/A	N/A
BMI	rs3817334	11	T	C	0.0265	0.004	5.15E-17	Yes	N/A	N/A
BMI	rs3849570	3	A	C	0.0147	0.0044	2.60E-08	No	rs11710970	0.91
BMI	rs3888190	16	A	C	0.0275	0.004	3.14E-23	Yes	N/A	N/A
BMI	rs4256980	11	G	C	0.0232	0.0041	2.90E-11	Yes	N/A	N/A
BMI	rs4740619	9	T	C	0.0157	0.0039	4.56E-09	Yes	N/A	N/A
BMI	rs543874	1	G	A	0.0603	0.005	2.62E-35	Yes	N/A	N/A
BMI	rs6477694	9	C	T	0.0211	0.0041	2.67E-08	Yes	N/A	N/A
BMI	rs6567160	18	C	T	0.0563	0.0046	3.93E-53	Yes	N/A	N/A
BMI	rs657452	1	A	G	0.0233	0.0041	5.48E-13	Yes	N/A	N/A
BMI	rs6804842	3	G	A	0.017	0.004	2.48E-09	Yes	N/A	N/A
BMI	rs7138803	12	A	G	0.0348	0.0041	8.15E-24	Yes	N/A	N/A
BMI	rs7141420	14	T	C	0.0262	0.0039	1.23E-14	Yes	N/A	N/A
BMI	rs7243357	18	T	G	0.0176	0.0051	3.86E-08	Yes	N/A	N/A
BMI	rs758747	16	T	C	0.0193	0.0047	7.47E-10	Yes	N/A	N/A
BMI	rs7599312	2	G	A	0.0183	0.0044	1.17E-10	Yes	N/A	N/A
BMI	rs7899106	10	G	A	0.0439	0.0091	2.96E-08	Yes	N/A	N/A
BMI	rs7903146	10	C	T	0.018	0.0044	1.11E-11	Yes	N/A	N/A
BMI	rs9400239	6	C	T	0.0225	0.0043	1.61E-08	Yes	N/A	N/A
BMI	rs9925964	16	A	G	0.0196	0.0041	8.11E-10	Yes	N/A	N/A
WHR	rs1011731	1	G	A	0.024	0.004	1.07E-08	Yes	N/A	N/A
WHR	rs10195252	2	T	C	0.044	0.004	2.57E-09	Yes	N/A	N/A
WHR	rs10245353	7	A	C	0.034	0.006	1.57E-10	Yes	N/A	N/A
WHR	rs11048470	12	T	G	0.033	0.005	6.33E-12	Yes	N/A	N/A
WHR	rs1121980	16	A	G	0.038	0.005	1.33E-38	Yes	N/A	N/A
WHR	rs11663816	18	C	T	0.031	0.005	2.65E-11	Yes	N/A	N/A
WHR	rs12549058	8	G	T	0.046	0.009	3.17E-10	No	rs16937647	0.92
WHR	rs1294421	6	G	T	0.033	0.005	6.93E-14	Yes	N/A	N/A
WHR	rs1358980	6	T	C	0.049	0.005	1.98E-14	Yes	N/A	N/A
WHR	rs1440372	15	C	T	0.021	0.005	7.59E-09	Yes	N/A	N/A

WHR	rs1443512	12	A	C	0.038	0.005	2.76E-11	Yes	N/A	N/A
WHR	rs1569135	2	A	G	0.022	0.004	1.00E-12	Yes	N/A	N/A
WHR	rs16996700	20	T	C	0.022	0.005	1.60E-08	Yes	N/A	N/A
WHR	rs17109256	14	A	G	0.018	0.005	3.022E-08	Yes	N/A	N/A
WHR	rs17451107	3	T	C	0.021	0.005	3.50E-11	Yes	N/A	N/A
WHR	rs2075650	19	A	G	0.031	0.007	6.43E-09	Yes	N/A	N/A
WHR	rs2179129	22	A	G	0.026	0.005	1.24E-09	Yes	N/A	N/A
WHR	rs2287019	19	C	T	0.019	0.006	4.344E-09	No	rs11672660	0.89
WHR	rs2765539	1	T	C	0.031	0.005	1.08E-12	Yes	N/A	N/A
WHR	rs3786897	19	G	A	0.021	0.005	3.95E-11	Yes	N/A	N/A
WHR	rs459193	5	A	G	0.027	0.005	6.02E-12	Yes	N/A	N/A
WHR	rs4640244	17	G	A	0.025	0.005	3.11E-08	Yes	N/A	N/A
WHR	rs4846565	1	G	A	0.048	0.005	4.75E-11	Yes	N/A	N/A
WHR	rs4929927	11	G	A	0.022	0.005	7.60E-09	Yes	N/A	N/A
WHR	rs7801581	7	T	C	0.022	0.006	4.951E-08	Yes	N/A	N/A
WHR	rs929641	2	A	G	0.022	0.004	4.25E-09	Yes	N/A	N/A
WHR	rs9491696	6	G	C	0.044	0.004	4.88E-30	Yes	N/A	N/A
WHR	rs9860730	3	A	G	0.042	0.005	2.84E-10	Yes	N/A	N/A

**Instrument**	**SNP ID**	**Chr**	**Effect allele**	**Other allele**	**Beta***	**SE**	***p-value***	***Included in adiposity GWAS***		***Proxy SNP***		***LD R^2***	
								BMI GWAS	***WHR GWAS***	***BMI GWAS***	***WHR GWAS***	***BMI GWAS***	***WHR GWAS***
CRP	rs10089518	8	C	T	0.025746	0.003807	1.39E-11	Yes	Yes	N/A	N/A	N/A	N/A
CRP	rs10240168	7	C	G	0.028684	0.004343	4.11E-11	Yes	Yes	N/A	N/A	N/A	N/A
CRP	rs10512597	17	C	T	0.036931	0.00489	4.44E-14	Yes	Yes	N/A	N/A	N/A	N/A
CRP	rs1051338	10	G	T	0.023881	0.003992	2.27E-09	Yes	Yes	N/A	N/A	N/A	N/A
CRP	rs10521222	16	C	T	0.104411	0.010714	2.06E-22	Yes	No	N/A	None available	N/A	N/A
CRP	rs10778215	12	T	A	0.033242	0.003583	1.86E-20	Yes	Yes	N/A	N/A	N/A	N/A
CRP	rs10832027	11	A	G	0.025944	0.003745	4.43E-12	Yes	Yes	N/A	N/A	N/A	N/A

CRP	rs10925027	1	T	C	0.036035	0.00382	4.25E-21	Yes	Yes	N/A	N/A	N/A	N/A
CRP	rs11108056	12	C	G	0.027907	0.003708	5.42E-14	Yes	Yes	N/A	N/A	N/A	N/A
CRP	rs11615578	12	T	C	0.047139	0.004401	9.8E-27	Yes	Yes	N/A	N/A	N/A	N/A
CRP	rs11783705	8	C	A	0.032575	0.00428	2.85E-14	Yes	Yes	N/A	N/A	N/A	N/A
CRP	rs12043437	1	T	C	0.04123	0.005901	2.91E-12	Yes	Yes	N/A	N/A	N/A	N/A
CRP	rs12202641	6	C	T	0.022804	0.003617	3E-10	Yes	Yes	N/A	N/A	N/A	N/A
CRP	rs12310160	12	C	T	0.051711	0.005215	3.82E-23	Yes	Yes	N/A	N/A	N/A	N/A
CRP	rs12587622	14	G	A	0.020798	0.003609	8.52E-09	Yes	Yes	N/A	N/A	N/A	N/A
CRP	rs1260326	2	T	C	0.073462	0.003604	2.72E-92	Yes	Yes	N/A	N/A	N/A	N/A
CRP	rs12960928	18	C	T	0.024	0.003993	1.91E-09	Yes	Yes	N/A	N/A	N/A	N/A
CRP	rs12995480	2	C	T	0.031261	0.004855	1.24E-10	Yes	Yes	N/A	N/A	N/A	N/A
CRP	rs13030345	2	T	G	0.052982	0.004989	2.6E-26	Yes	Yes	N/A	N/A	N/A	N/A
CRP	rs13233571	7	C	T	0.056895	0.005476	2.95E-25	Yes	Yes	N/A	N/A	N/A	N/A
CRP	rs13282752	8	A	G	0.036001	0.004433	4.85E-16	Yes	Yes	N/A	N/A	N/A	N/A
CRP	rs13409371	2	A	G	0.048232	0.003847	5.07E-36	Yes	Yes	N/A	N/A	N/A	N/A
CRP	rs1371614	2	C	T	0.026321	0.004234	5.23E-10	Yes	Yes	N/A	N/A	N/A	N/A
CRP	rs1441169	2	A	G	0.024926	0.003725	2.27E-11	Yes	Yes	N/A	N/A	N/A	N/A
CRP	rs1490384	6	C	T	0.024816	0.003545	2.65E-12	Yes	Yes	N/A	N/A	N/A	N/A
CRP	rs1509394	2	T	C	0.025685	0.004147	6.05E-10	Yes	Yes	N/A	N/A	N/A	N/A
CRP	rs1558902	16	A	T	0.033926	0.003701	5.2E-20	Yes	Yes	N/A	N/A	N/A	N/A
CRP	rs1582763	11	G	A	0.022107	0.0037	2.37E-09	Yes	Yes	N/A	N/A	N/A	N/A
CRP	rs1616534	8	T	C	0.030177	0.003703	3.88E-16	Yes	Yes	N/A	N/A	N/A	N/A
CRP	rs1659685	2	T	C	0.026139	0.003886	1.8E-11	Yes	Yes	N/A	N/A	N/A	N/A
CRP	rs17149760	8	A	G	0.038914	0.004292	1.31E-19	Yes	Yes	N/A	N/A	N/A	N/A
CRP	rs17616063	16	A	G	0.106356	0.011169	1.8E-21	Yes	No	N/A	None available	N/A	N/A
CRP	rs17643262	19	G	A	0.033345	0.005874	1.41E-08	Yes	Yes	N/A	N/A	N/A	N/A
CRP	rs17658229	5	C	T	0.055568	0.009522	5.5E-09	Yes	Yes	N/A	N/A	N/A	N/A
CRP	rs17759740	2	T	A	0.033895	0.005937	1.16E-08	Yes	Yes	N/A	N/A	N/A	N/A
CRP	rs178810	17	T	C	0.02001	0.003606	2.95E-08	Yes	Yes	N/A	N/A	N/A	N/A

CRP	rs1800961	20	C	T	0.1115	0.011267	4.63E-23	Yes	Yes	N/A	N/A	N/A	N/A
CRP	rs1805096	1	G	A	0.104381	0.003614	2.17E-183	Yes	Yes	N/A	N/A	N/A	N/A
CRP	rs1880241	7	A	G	0.027537	0.003687	8.41E-14	Yes	Yes	N/A	N/A	N/A	N/A
CRP	rs1985096	19	A	T	0.042135	0.006467	7.48E-11	Yes	Yes	N/A	N/A	N/A	N/A
CRP	rs2064009	8	T	C	0.027111	0.003549	2.28E-14	Yes	Yes	N/A	N/A	N/A	N/A
CRP	rs206967	12	C	T	0.027577	0.004797	9.2E-09	Yes	Yes	N/A	N/A	N/A	N/A
CRP	rs2239222	14	G	A	0.035484	0.003901	9.87E-20	Yes	Yes	N/A	N/A	N/A	N/A
CRP	rs2293476	1	C	G	0.030262	0.004225	8.27E-13	Yes	Yes	N/A	N/A	N/A	N/A
CRP	rs2315008	20	G	T	0.023467	0.003777	5.36E-10	Yes	Yes	N/A	N/A	N/A	N/A
CRP	rs2352975	3	C	T	0.024897	0.004026	6.43E-10	Yes	Yes	N/A	N/A	N/A	N/A
CRP	rs2572397	8	A	G	0.025043	0.003635	5.85E-12	Yes	Yes	N/A	N/A	N/A	N/A
CRP	rs2686343	12	A	G	0.023413	0.003749	4.35E-10	Yes	Yes	N/A	N/A	N/A	N/A
CRP	rs2710804	7	C	T	0.021262	0.003737	0.000000013	Yes	Yes	N/A	N/A	N/A	N/A
CRP	rs2794520	1	C	T	0.182186	0.003712	1.02E-305	Yes	Yes	N/A	N/A	N/A	N/A
CRP	rs2798604	1	T	G	0.021868	0.003837	1.24E-08	Yes	Yes	N/A	N/A	N/A	N/A
CRP	rs2801578	1	T	A	0.04819	0.003913	8.09E-35	Yes	Yes	N/A	N/A	N/A	N/A
CRP	rs2836878	21	G	A	0.042902	0.004079	7.71E-26	Yes	Yes	N/A	N/A	N/A	N/A
CRP	rs2852151	18	A	G	0.024735	0.003655	1.36E-11	Yes	Yes	N/A	N/A	N/A	N/A
CRP	rs2891677	8	T	C	0.019859	0.003511	1.59E-08	Yes	Yes	N/A	N/A	N/A	N/A
CRP	rs2948305	8	A	G	0.023948	0.003778	2.39E-10	Yes	Yes	N/A	N/A	N/A	N/A
CRP	rs2965109	19	T	C	0.025284	0.00414	1.04E-09	Yes	Yes	N/A	N/A	N/A	N/A
CRP	rs3134899	6	T	C	0.023329	0.004274	4.93E-08	Yes	Yes	N/A	N/A	N/A	N/A
CRP	rs340005	15	A	G	0.030007	0.003736	1.01E-15	Yes	Yes	N/A	N/A	N/A	N/A
CRP	rs4075359	8	T	C	0.026545	0.003705	8.07E-13	Yes	Yes	N/A	N/A	N/A	N/A
CRP	rs4092465	18	G	A	0.027483	0.004364	3.11E-10	Yes	Yes	N/A	N/A	N/A	N/A
CRP	rs4129267	1	C	T	0.087519	0.003612	1.2E-129	Yes	Yes	N/A	N/A	N/A	N/A
CRP	rs4147730	11	G	A	0.027431	0.005016	4.65E-08	Yes	Yes	N/A	N/A	N/A	N/A
CRP	rs4246598	2	A	C	0.022063	0.003547	5.11E-10	Yes	Yes	N/A	N/A	N/A	N/A
CRP	rs4420638	19	A	G	0.229459	0.006122	2.26E-307	Yes	No	N/A	None available	N/A	N/A

CRP	rs4660443	1	T	C	0.028822	0.004684	7.8E-10	Yes	Yes	N/A	N/A	N/A	N/A
CRP	rs469772	1	C	T	0.031327	0.004542	5.54E-12	Yes	Yes	N/A	N/A	N/A	N/A
CRP	rs4767878	12	G	A	0.087865	0.01311	2.13E-11	Yes	No	N/A	None available	N/A	N/A
CRP	rs4774590	15	G	A	0.022169	0.003985	2.71E-08	Yes	Yes	N/A	N/A	N/A	N/A
CRP	rs4841132	8	G	A	0.065095	0.006243	2E-25	Yes	Yes	N/A	N/A	N/A	N/A
CRP	rs4841407	8	G	A	0.025494	0.003677	4.24E-12	Yes	Yes	N/A	N/A	N/A	N/A
CRP	rs541619	12	A	G	0.075692	0.004812	1.04E-55	Yes	Yes	N/A	N/A	N/A	N/A
CRP	rs6001193	22	A	G	0.027809	0.003706	6.53E-14	Yes	Yes	N/A	N/A	N/A	N/A
CRP	rs644234	9	G	T	0.022597	0.003708	1.13E-09	Yes	Yes	N/A	N/A	N/A	N/A
CRP	rs6485716	11	G	A	0.028949	0.005029	8.81E-09	Yes	Yes	N/A	N/A	N/A	N/A
CRP	rs6485751	11	G	C	0.03089	0.004359	1.43E-12	Yes	Yes	N/A	N/A	N/A	N/A
CRP	rs660895	6	G	A	0.034428	0.004525	2.89E-14	Yes	Yes	N/A	N/A	N/A	N/A
CRP	rs6672992	1	C	A	0.050172	0.005563	2E-19	Yes	Yes	N/A	N/A	N/A	N/A
CRP	rs680925	1	A	G	0.030454	0.003734	3.64E-16	Yes	Yes	N/A	N/A	N/A	N/A
CRP	rs7015461	8	T	C	0.028417	0.004287	3.5E-11	Yes	Yes	N/A	N/A	N/A	N/A
CRP	rs707939	6	A	C	0.024086	0.003858	4.44E-10	Yes	Yes	N/A	N/A	N/A	N/A
CRP	rs7310409	12	G	A	0.137075	0.003706	2.54E-299	Yes	Yes	N/A	N/A	N/A	N/A
CRP	rs7837587	8	C	T	0.026823	0.003537	3.53E-14	Yes	Yes	N/A	N/A	N/A	N/A
CRP	rs863018	1	C	T	0.043948	0.003941	7.56E-29	Yes	Yes	N/A	N/A	N/A	N/A
CRP	rs9284725	2	C	A	0.02731	0.00419	7.34E-11	Yes	Yes	N/A	N/A	N/A	N/A
CRP	rs937813	2	C	T	0.047625	0.006223	2.05E-14	Yes	Yes	N/A	N/A	N/A	N/A
CRP	rs9385532	6	C	T	0.02556	0.003804	1.9E-11	Yes	Yes	N/A	N/A	N/A	N/A
CRP	rs9929889	16	T	C	0.02528	0.004085	6.24E-10	Yes	Yes	N/A	N/A	N/A	N/A

*Where necessary, beta coefficients were multiplied by -1 to ensure all betas represented an increase in the respective traits and allele harmonisation was done to ensure alignment of alleles for both the SNP-X and SNP-Y associations. GWAS betas, SEs and p-values taken from Locke et al. 2015. Nature, 518(7538):197-206 (BMI); Shungin et al. 2015. Nature, 518(7538):187-96 (WHR); Ligthart et al. 2018. AJHG, 103(5): 691-706 (CRP); ☨proxy SNPs identified using online tool 'SNIPA' https://snipa.helmholtz-muenchen.de/snipa3/

Supplementary table 3. Sex-specific phenotypic correlations between adiposity indicators and CRP in UK Biobank participants (n= 367,583)

	CRP	
	Males	*Females*
**BMI**	0.1475	0.3198
**WHR**	0.1478	0.1988

Supplementary table 4. Direct, indirect and total effects of adiposity on grip strength estimated from structural equation models of observational data (n= 367,583)

	Difference (95% CI)* in grip strength (kg) by adiposity indicator			
	Males			
	BMI		WHR	
	Unadjusted	Adjusted**	Unadjusted	Adjusted**
Direct effect	0.66 (0.61, 0.70)	0.71 (0.67, 0.75)	-0.83 (-0.87, -0.79)	-0.31 (-0.35, -0.27)
Indirect effect	-0.38 (-0.40, 0.36)	-0.23 (-0.25, -0.22)	-0.21 (-0.23, -0.20)	-0.12 (-0.13, -0.10)
Total effect	0.28 (0.24,0.32)	0.48 (0.44, 0.51)	-1.04 (-1.08, -1.00)	-0.42 (-0.46, -0.39)
	Females			
	BMI		WHR	
	Unadjusted	Adjusted **	Unadjusted	Adjusted**
Direct effect	0.17 (0.13, 0.21)	0.32 (0.29, 0.36)	-0.45 (-0.48, -0.41)	-0.07 (-0.10, -0.04)
Indirect effect	-0.45 (-0.46, -0.43)	-0.26 (-0.27, -0.24)	-0.22 (-0.23, -0.21)	-0.11 (-0.12, -0.10)
Total effect	-0.27 (-0.30, -0.24)	0.07 (0.04, 0.09)	-0.67 (-0.70, -0.63)	-0.18 (-0.21, -0.15)

*associations represent estimated mean difference in grip strength (kg) per 1 SD higher adiposity indicator. **Models adjusted for the Townsend index of deprivation, smoking status, physical activity, alcohol intake, age at recruitment, and comorbidities (arthritis, asthma and depression).

Supplementary table 5. Estimates from univariable MR for total effect of adiposity* on grip strength (n= 367,583)

		**Difference (95% CI) in grip strength (kg) by adiposity indicator**	
		Males	
		BMI	WHR
Number of SNPs		76	28
IVW		0.50 (-0.01, 1.01)	0.25 (-0.80, 1.31)
	I^2	0.71	0.73
	Cochran's Q-statistic	257.0 (*p*<0.001)	98.1 (*p*<0.001)
WME		0.50 (0.01, 0.99)	-0.04 (-1.05, 0.97)
MR-Egger		0.05 (-1.17, 1.27)	0.61 (-1.79, 3.01)
	p-pleiotropy	0.43	0.74
	I^2_{GX}	0.82	0.72
		**Difference (95% CI) in grip strength (kg) by adiposity indicator**	
		Females	
		BMI	WHR
Number of SNPs		76	28
IVW		0.08 (-0.26, 0.43)	0.67 (0.30, 1.04)
	I^2	0.74	0.52
	Cochran's Q-statistic	285.1 (*p*<0.001)	56.5 (*p*<0.001)
WME		0.23 (-0.08, 0.54)	0.94 (0.56, 1.31)
MR-Egger		-0.02 (-0.84, 0.81)	2.01 (0.86, 3.16)
	p-pleiotropy	0.79	0.02
	I^2_{GX}	0.84	0.77

IVW: Inverse-variance-weighted; WME: weighted median estimator; MR-Egger: Mendelian randomisation Egger regression; *associations represent estimated mean difference in grip strength (kg) per 1 SD higher genetically predicted adiposity indicator; p-pleiotropy: p-value for overall horizontal pleiotropic effect indicated by the intercept from MR-Egger regression

Supplementary table 6. Total, direct and indirect effects of 1 SD increase in adiposity trait on grip strength (kg) after accounting for effects of CRP in MVMR (n= 367,583)

			**Males**			
		BMI			WHR	
Number of SNPs		164 (76 + 88)			112 (28 + 84)	
Conditional F-statistic		20.06			6.05	
	*Total effect**	*Direct effect*	*Indirect effect†*	*Total effect**	*Direct effect*	*Indirect effect†*
MVMR-IVW	0.50 (-0.01, 1.01)	0.76 (0.30, 1.21)	-0.26 (-0.95, 0.43)	0.25 (-0.80, 1.31)	0.32 (-0.55, 1.20)	-0.07 (-1.44, 1.30)
Q_A-heterogeneity		537.7 (*p*<0.001)			401.0 (*p*<0.001)	
MVMR-Egger	0.05 (-1.17, 1.27)	0.56 (-0.11, 1.24)	-0.51 (-1.91, 0.89)	0.61 (-1.79, 3.01)	0.76 (-0.44, 1.95)	-0.15 (-2.83, 2.53)
p-pleiotropy		0.46			0.29	
Q_A-heterogeneity		540.1 (*p*<0.001)			398.7 (*p*<0.001)	
MVMR-IVW (Q_{het})	-	0.61 (-0.16, 1.15)	-	-	0.75 (-0.25, 1.86)	-
			**Females**			
		BMI			WHR	
Number of SNPs		164 (76 + 88)			112 (28 + 84)	
Conditional F-statistic		21.86			12.04	
	*Total effect**	*Direct effect*	*Indirect effect†*	*Total effect**	*Direct effect*	*Indirect effect†*
MVMR-IVW	0.08 (-0.26, 0.43)	0.15 (-0.15, 0.46)	-0.07 (-0.53, 0.39)	0.67 (0.30, 1.04)	0.49 (0.08, 0.91)	0.18 (-0.38, 0.74)
Q_A-heterogeneity		674.8 (*p*<0.001)			331.1 (*p*<0.001)	
MVMR-Egger	-0.02 (-0.84, 0.81)	0.07 (-0.38, 0.52)	-0.09 (-1.03, 0.85	2.01 (0.86, 3.16)	1.25 (0.72, 1.78)	0.76 (-0.51, 2.03)
p-pleiotropy		0.62			<0.001	
Q_A-heterogeneity		675.2 (*p*<0.001)			290.8 (*p*<0.001)	
MVMR-IVW (Q_{het})	-	0.22 (-0.31, 0.53)	-	-	0.52 (0.11, 0.85)	-

MVMR-IVW: Multivariable Inverse-variance-weighted; MVMR-Egger: Multivariable MR Egger regression; MVMR-IVW (Q_{het}): modified version of MR-IVW accounting for excess heterogeneity; *associations are from the equivalent univariable MR and represent estimated mean difference in grip strength (kg) per 1 SD higher genetically predicted adiposity indicator; p-pleiotropy: p-value for overall horizontal pleiotropic effect indicated by the intercept from MVMR-Egger regression; †estimated using the 'difference method' with 95% CIs estimated using the 'propagation of errors method'(12)

Supplementary table 7. Estimates from univariable MR for total effect of adiposity* on grip strength (kg) after removing adiposity SNPs associated with potential confounders (n= 367,583)

	Difference (95% CI) in grip strength (kg) by adiposity indicator	
	Males	
	BMI	WHR
Number of SNPs removed	19	7
IVW	0.74 (0.13, 1.35)	-0.07 (-1.40, 1.26)
I^2	0.66	0.73
Cochran's Q-statistic	163.1 (*p*<0.001)	73.8 (*p*<0.001)
WME	1.04 (0.44, 1.64)	-0.51 (-1.74, 0.73)
MR-Egger	0.65 (-1.24, 2.53)	-1.59 (-5.27, 2.10)
p-pleiotropy	0.92	0.39
I^2_{GX}	0.48	0.53
	**Difference (95% CI) in grip strength (kg) by adiposity indicator**	
	Females	
	BMI	WHR
Number of SNPs removed	27	6
IVW	0.23 (-0.20, 0.65)	0.70 (0.27, 1.12)
I^2	0.59	0.54
Cochran's Q-statistic	118.3 (*p*<0.001)	45.2 (*p*=0.002)
WME	0.28 (-0.16, 0.72)	0.99 (0.52, 1.45)
MR-Egger	-0.63 (-1.91, 0.65)	1.93 (0.67, 3.20)
p-pleiotropy	0.16	0.04
I^2_{GX}	0.49	0.80

IVW: Inverse-variance-weighted; WME: weighted median estimator; MR-Egger: Mendelian randomisation Egger regression; *associations represent estimated mean difference in grip strength (kg) per 1 SD higher genetically predicted adiposity indicator; p-pleiotropy: p-value for overall horizontal pleiotropic effect indicated by the intercept from MR-Egger regression

Supplementary table 8. Estimates from univariable MR for total effect of WHR on grip strength (kg) in females after removing three SNPs of high influence (n= 197, 408)

	Difference (95% CI) in grip strength (kg)
Number of SNPs	25
IVW	0.78 (0.48, 1.08)
I^2	0.22
Cochran's Q-statistic	30.8 (0.16)
WME	0.94 (0.53, 1.35)
MR-Egger	1.74 (0.75, 2.74)
p-pleiotropy	0.05
I^2_{GX}	0.77

IVW: Inverse-variance-weighted; WME: weighted median estimator; MR-Egger: Mendelian randomisation Egger regression; *associations represent estimated mean difference in grip strength (kg) per 1 SD higher genetically predicted WHR; p-pleiotropy: p-value for overall horizontal pleiotropic effect indicated by the intercept from MR-Egger regression

Supplementary table 9. Total, direct and indirect effects of 1 SD increase in adiposity trait on grip strength (kg) after removing adiposity SNPs associated with potential confounders (n= 367,583)

			**Males**			
		BMI			WHR	
Number of SNPs		145 (57 + 88)			105 (21 + 84)	
Conditional F-statistic		13.35			5.00	
	*Total effect**	*Direct effect*	*Indirect effect†*	*Total effect**	*Direct effect*	*Indirect effect†*
MVMR-IVW	0.74 (0.13, 1.35)	1.16 (0.60, 1.72)	-0.42 (-1.26, 0.41)	-0.07 (-1.40, 1.26)	0.11 (-0.88, 1.10)	-0.18 (-1.84, 1.48)
Q_A-heterogeneity		435.9 (*p*<0.001)			375.2 (*p*<0.001)	
MVMR-Egger	0.65 (-1.24, 2.53)	1.21 (0.32, 2.10)	-0.56 (-2.65, 1.53)	-1.59 (-5.27, 2.10)	0.44 (-0.94, 1.82)	-2.03 (-5.96, 1.90)
p-pleiotropy		0.88			0.50	
Q_A-heterogeneity		433.1 (*p*<0.001)			373.6 (*p*<0.001)	
MVMR-IVW (Q_{het})	-	1.34 (0.91, 2.13)	-	-	0.50 (-1.01, 1.68)	-
			**Females**			
		BMI			WHR	
Number of SNPs		137 (49 + 88)			106 (22 + 84)	
Conditional F-statistic		12.66			10.83	
	*Total effect**	*Direct effect*	*Indirect effect†*	*Total effect**	*Direct effect*	*Indirect effect†*
MVMR-IVW	0.23 (-0.20, 0.65)	0.26 (-0.10, 0.62)	-0.03 (-0.58, 0.52)	0.70 (0.27, 1.12)	0.44 (-0.01, 0.90)	0.26 (-0.37, 0.89)
Q_A-heterogeneity		403.1 (*p*<0.001)			321.8 (*p*<0.001)	
MVMR-Egger	-0.63 (-1.91, 0.65)	0.10 (-0.43, 0.62)	-0.73 (-2.11, 0.65)	1.93 (0.67, 3.20)	1.22 (0.64, 1.79)	0.71 (-0.68, 2.10)
p-pleiotropy		0.40			<0.001	
Q_A-heterogeneity		399.5 (*p*<0.001)			279.8 (*p*<0.001)	
MVMR-IVW (Q_{het})	-	0.34 (-0.17, 0.70)	-	-	0.44 (-0.08, 0.81)	-

MVMR-IVW: Multivariable Inverse-variance-weighted; MVMR-Egger: Multivariable MR Egger regression; MVMR-IVW (Q_{het}): modified version of MR-IVW accounting for excess heterogeneity; *associations are from the equivalent univariable MR and represent estimated mean difference in grip strength (kg) per 1 SD higher genetically predicted adiposity indicator; p-pleiotropy: p-value for overall horizontal pleiotropic effect indicated by the intercept from MVMR-Egger regression; †estimated using the 'difference method' with 95% CIs estimated using the 'propagation of errors method'(12)

Supplementary table 10. Direct effect estimates from multivariable MR for total effect of WHR on grip strength (kg) in females after removing three SNPs of high influence (n= 197, 408)

		Mean difference (95% CI) in grip strength (kg)
Number of SNPs		109 (25 WHR+ 84 CRP)
IVW		0.58 (0.16, 0.99)
	Q_A-*heterogeneity*	312.9 (*p*<0.001)
MR-Egger		1.31 (0.79, 1.83)
	p-pleiotropy	<0.001
	Q_A-*heterogeneity*	268.7 (*p*<0.001)
MVMR-IVW (Q_{-het})		0.59 (0.18, 0.89)

MVMR-IVW: Multivariable Inverse-variance-weighted; MVMR-Egger: Multivariable MR Egger regression; MVMR-IVW (Q_{-het}): modified version of MR-IVW accounting for excess heterogeneity; *associations represent estimated mean difference in grip strength (kg) per 1 SD higher genetically predicted WHR; p-pleiotropy: p-value for overall horizontal pleiotropic effect indicated by the intercept from MVMR-Egger regression

Supplementary figure 1. Directed acyclic graph showing tested relationship between adiposity and grip strength

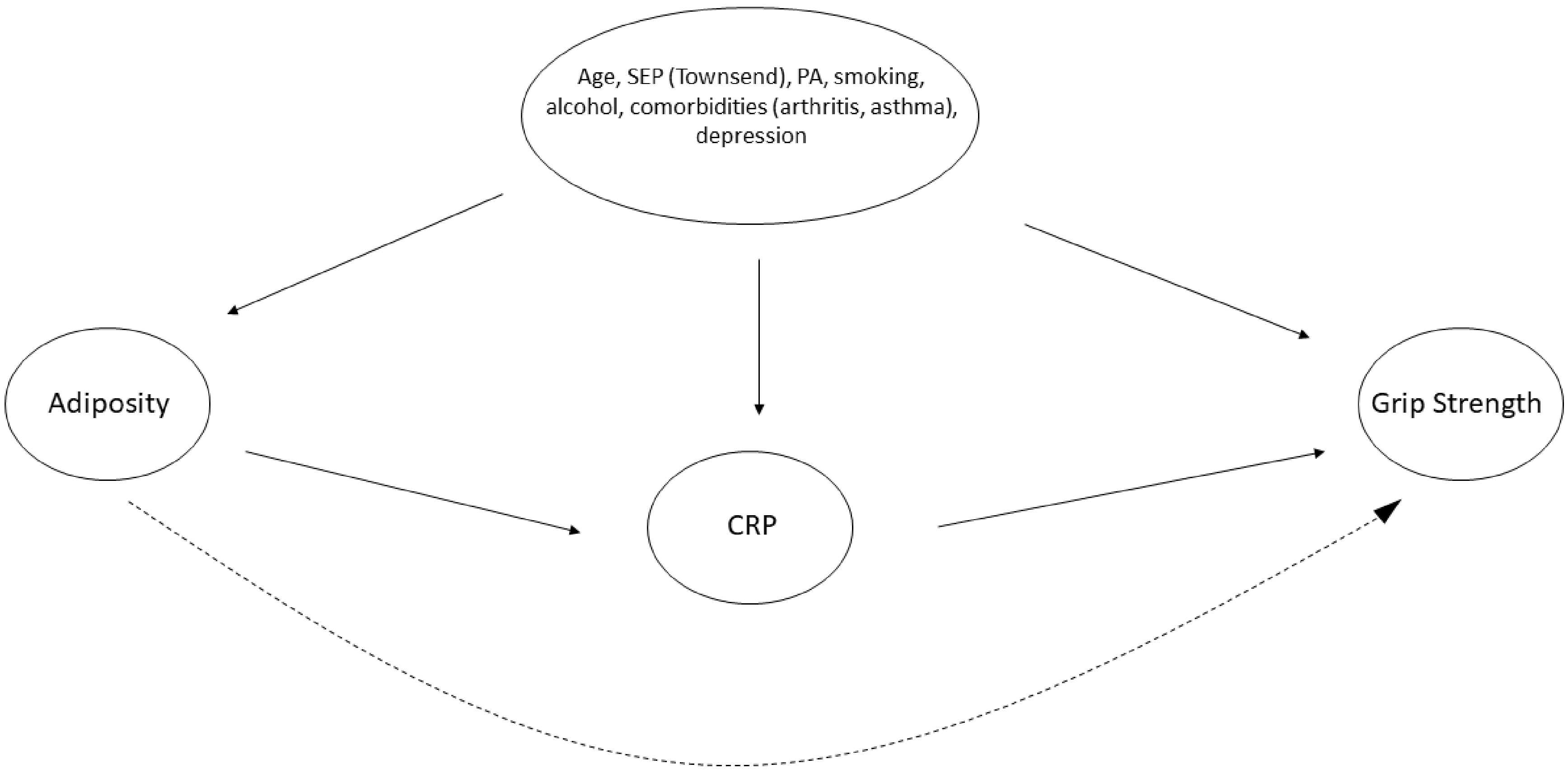

Supplementary Figure 2. Funnel plot of risk estimates of BMI SNPs on grip strength against instrumental strength: Males

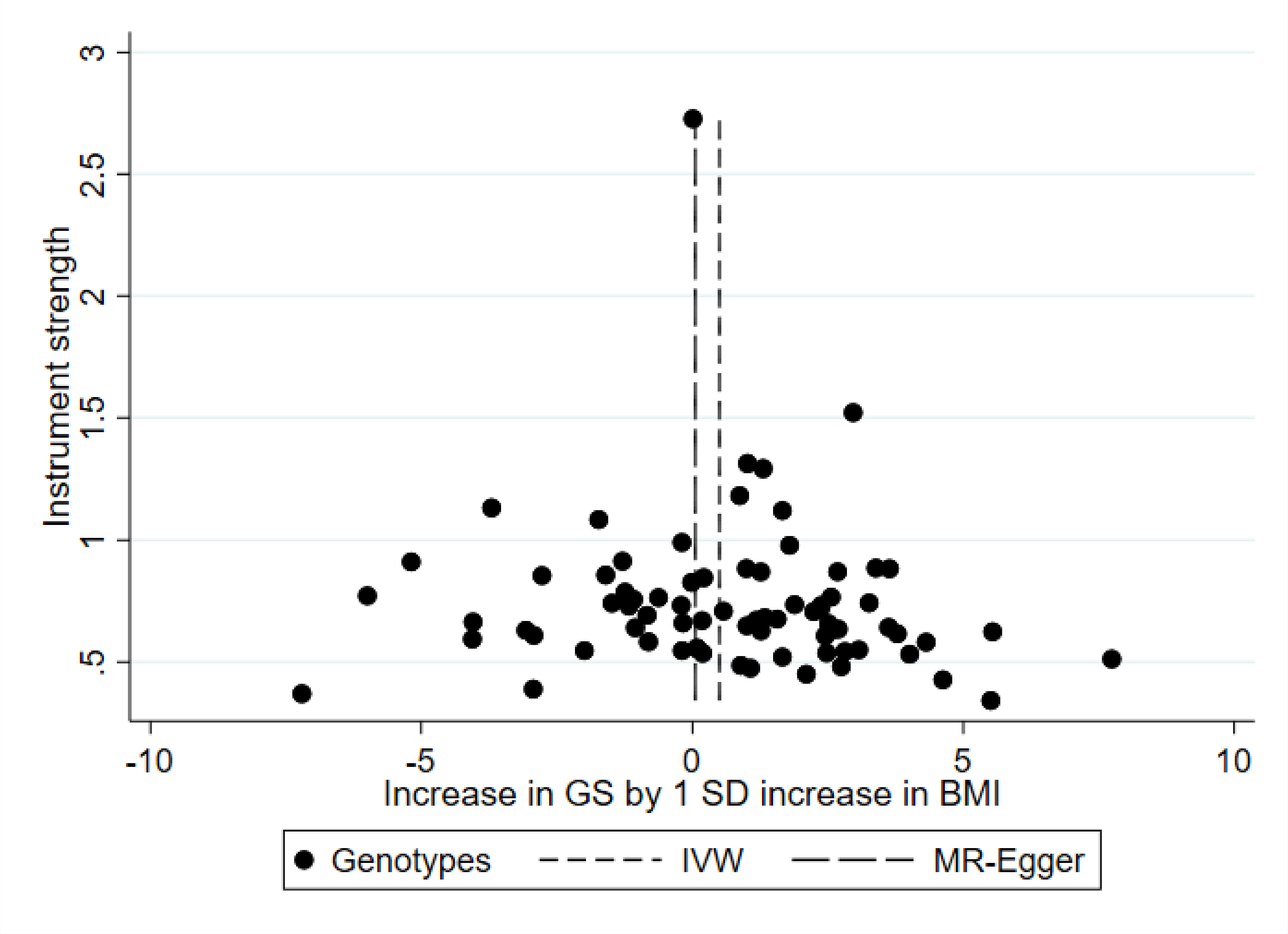

Supplementary figure 3. Funnel plot of risk estimates of BMI SNPs on grip strength against instrumental strength: Females

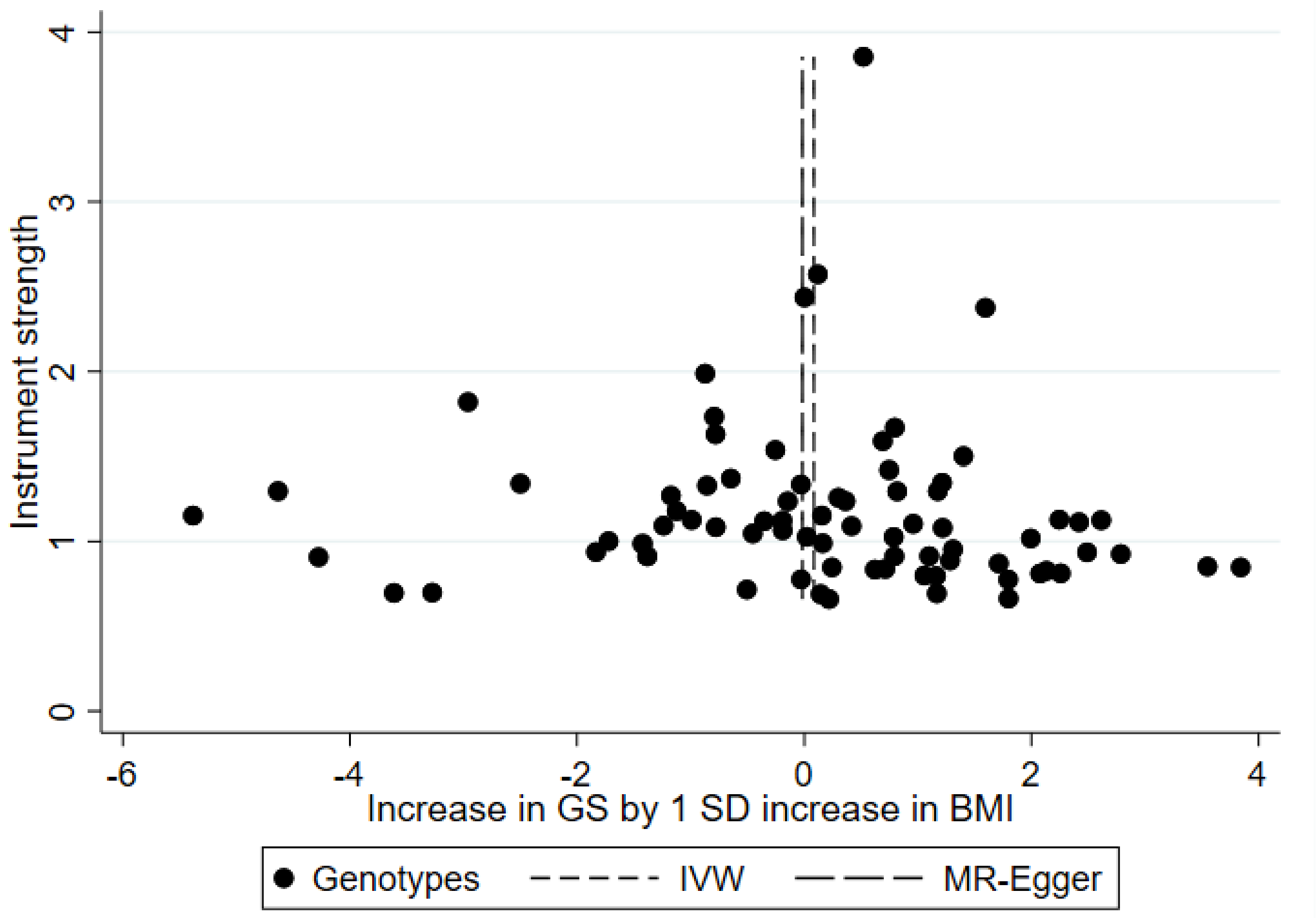

Supplementary figure 4. Funnel plot of risk estimates of WHR SNPs on grip strength against instrumental strength: Males

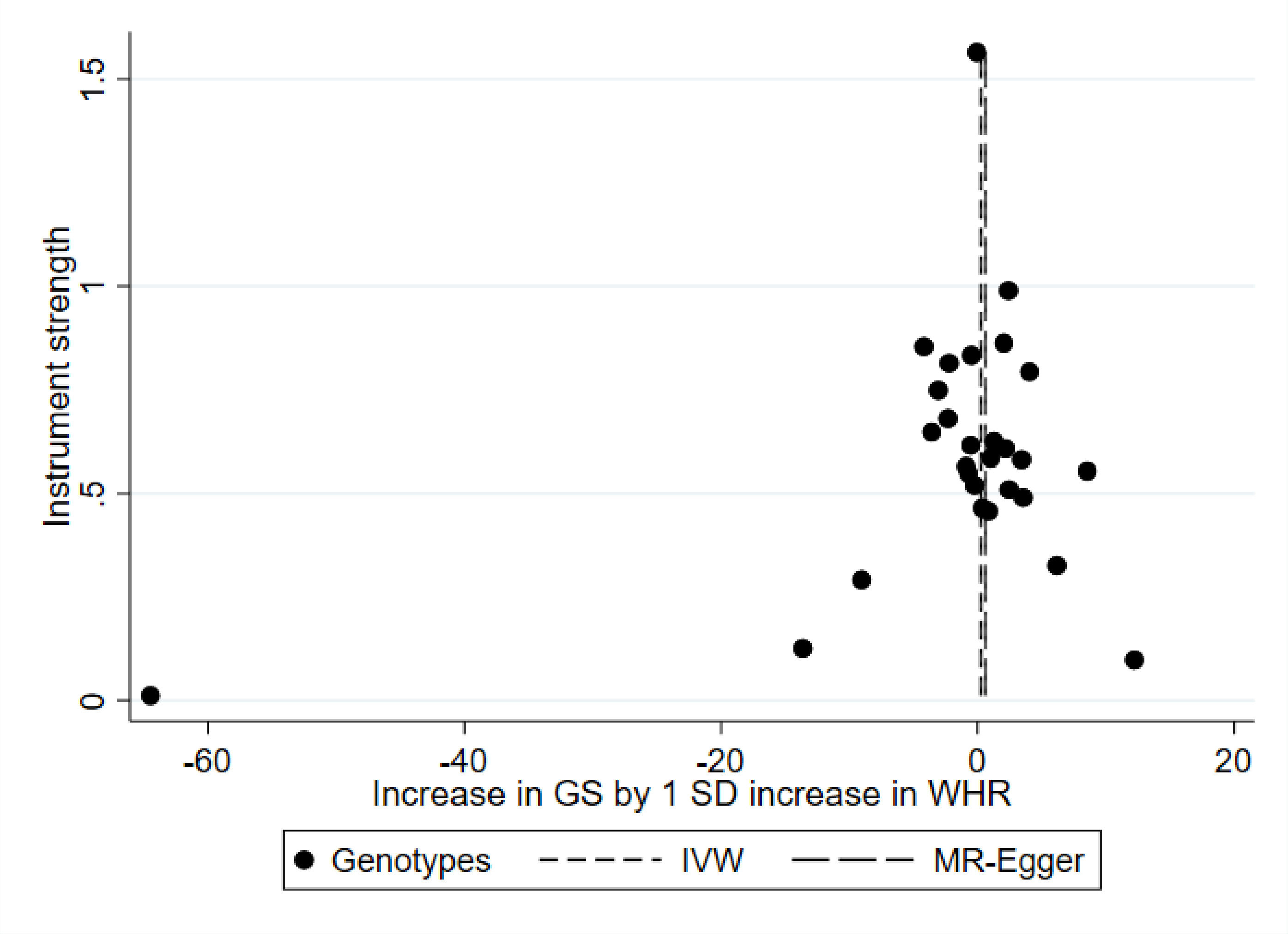

Supplementary figure 5. Funnel plot of risk estimates of WHR SNPs on grip strength against instrumental strength: Females

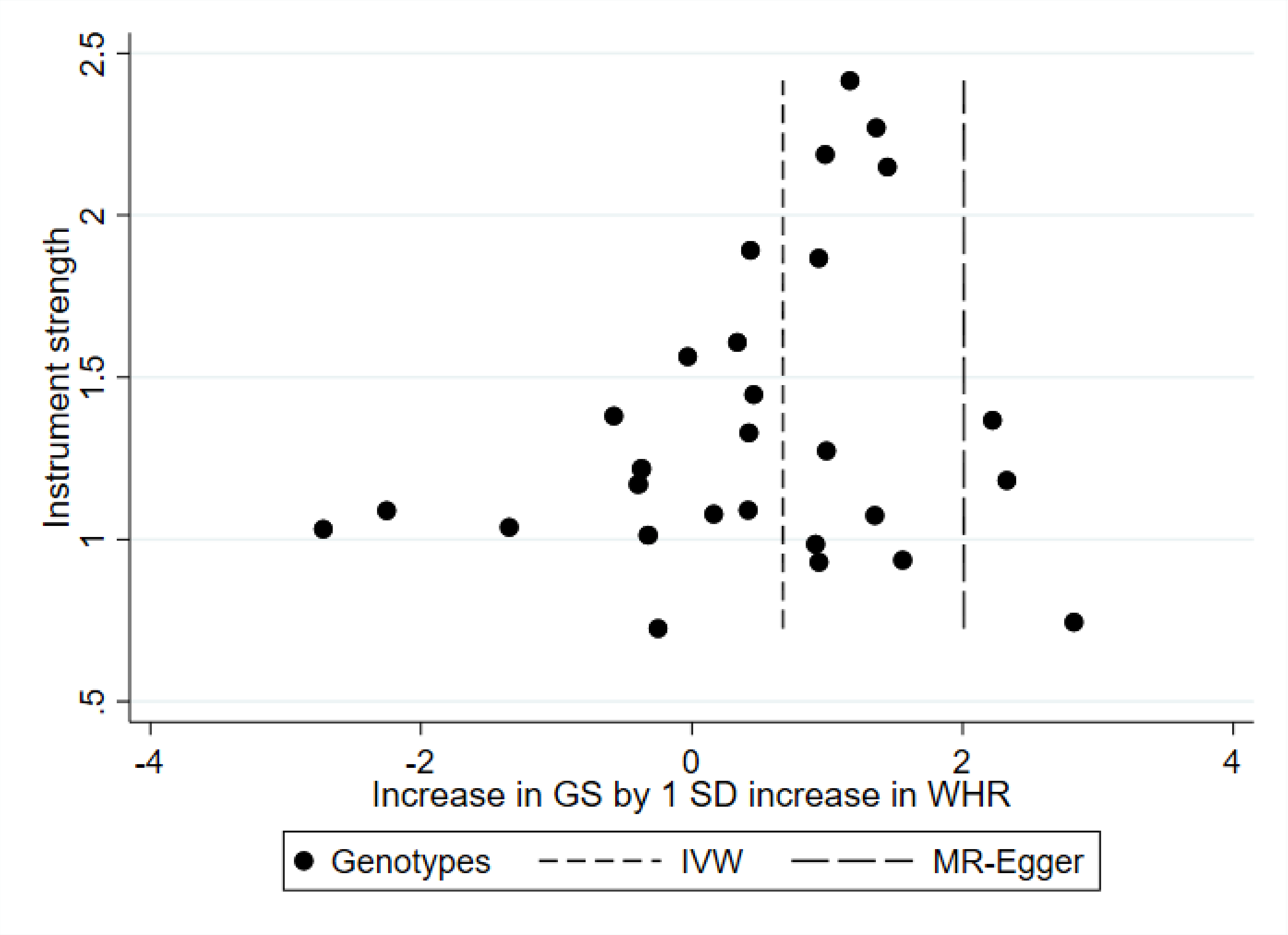

References

1. Bycroft C, Freeman C, Petkova D, Band G, Elliott LT, Sharp K, et al. The UK Biobank resource with deep phenotyping and genomic data. Nature. 2018;562(7726):203-9.
2. Davey Smith G, Hemani G. Mendelian randomization: genetic anchors for causal inference in epidemiological studies. Human molecular genetics. 2014;23(R1):R89-R98.
3. Burgess S, Bowden J. Integrating summarized data from multiple genetic variants in Mendelian randomization: bias and coverage properties of inverse-variance weighted methods. arXiv preprint arXiv:151204486. 2015.
4. Burgess S, Bowden J, Fall T, Ingelsson E, Thompson SG. Sensitivity analyses for robust causal inference from Mendelian randomization analyses with multiple genetic variants. Epidemiology (Cambridge, Mass). 2017;28(1):30.
5. Bowden J, Del Greco M F, Minelli C, Davey Smith G, Sheehan N, Thompson J. A framework for the investigation of pleiotropy in two-sample summary data Mendelian randomization. Statistics in medicine. 2017;36(11):1783-802.
6. Bowden J, Davey Smith G, Haycock PC, Burgess S. Consistent estimation in Mendelian randomization with some invalid instruments using a weighted median estimator. Genetic epidemiology. 2016;40(4):304-14.
7. Burgess S, Bowden J, Dudbridge F, Thompson SG. Robust instrumental variable methods using multiple candidate instruments with application to Mendelian randomization. arXiv preprint arXiv:160603729. 2016.
8. Sanderson E, Windmeijer F. A weak instrument F-test in linear IV models with multiple endogenous variables. Journal of econometrics. 2016;190(2):212-21.
9. Sanderson E, Davey Smith G, Windmeijer F, Bowden J. An examination of multivariable Mendelian randomization in the single-sample and two-sample summary data settings. International journal of epidemiology. 2019;48(3):713-27.
10. Cragg JG, Donald SG. Testing identifiability and specification in instrumental variable models. Econometric Theory. 1993;9(2):222-40.
11. Rees JM, Wood AM, Burgess S. Extending the MR-Egger method for multivariable Mendelian randomization to correct for both measured and unmeasured pleiotropy. Statistics in medicine. 2017;36(29):4705-18.
12. Burgess S, Thompson DJ, Rees JM, Day FR, Perry JR, Ong KK. Dissecting causal pathways using Mendelian randomization with summarized genetic data: application to age at menarche and risk of breast cancer. Genetics. 2017;207(2):481-7.
